# Development and equivalence of new faces for inclusion in the Childhood Asthma Control Test (C-ACT) response scale

**DOI:** 10.1186/s41687-021-00390-2

**Published:** 2021-11-06

**Authors:** Kate Sully, Nicola Bonner, Helena Bradley, Robyn von Maltzahn, Rob Arbuckle, Louise Walker-Nthenda, Aoife Mahon, Brandon Becker, Louise O’Hara, Katherine B. Bevans, Mark Kosinski, Robert S. Zeiger, Ross Mackenzie, Linda Nelsen

**Affiliations:** 1 Patient-Centered Outcomes, Adelphi Values, Bollington, Cheshire, UK; 2 Patient Centered Outcomes, Value Evidence Outcomes, GlaxoSmithKline, 980 Great West Road, Brentford, Middlesex, TW8 9GS UK; 3 Value Evidence Outcomes, GlaxoSmithKline, Stevenage, UK; 4 World Wide Health Economics and Outcomes Research, Bristol Myers Squibb, Lawrenceville, NJ USA; 5 Temple University, Philadelphia, PA USA; 6 QualityMetric Incorporated, LLC, 1301 Atwood Ave., Johnston, RI USA; 7 Allergy Department, Kaiser Permanente Southern California, San Diego, CA USA; 8 North Staffordshire Combined Healthcare NHS Trust, Staffordshire, UK; 9 Patient Centered Outcomes, VEO, GSK, Collegeville, PA USA

**Keywords:** Pediatric asthma, Qualitative equivalence, CHILDHOOD ASTHMA CONTROL TEST, Patient-reported outcome, Asthma control, Clinical outcome assessment, Image-based response options, Asthma symptoms

## Abstract

**Background:**

Accurate symptom monitoring is vital when managing pediatric asthma, providing an opportunity to improve control and relieve associated burden. The CHILDHOOD ASTHMA CONTROL TEST (C-ACT) has been validated for asthma control assessment in children; however, there are concerns that response option images used in the C-ACT are not culturally universal and could be misinterpreted. This cross-sectional, qualitative study developed and evaluated alternative response option images using interviews with children with asthma aged 4–11 years (and their parents/caregivers) in the United States, Spain, Poland, and Argentina. Interviews were conducted in two stages (with expert input) to evaluate the appropriateness, understanding and qualitative equivalence of the alternative images (both on paper and electronically). This included comparing the new images with the original C-ACT response scale, to provide context for equivalence results.

**Results:**

Alternative response option images included scale A (simple faces), scale B (circles of decreasing size), and scale C (squares of decreasing quantity). In Stage 1, most children logically ranked images using scales A, B and C (66.7%, 79.0% and 70.6%, respectively). However, some children ranked the images in scales B (26.7%) and C (58.3%) in reverse order. Slightly more children could interpret the images within the context of their asthma in scale B (68.4%) than A (55.6%) and C (47.5%). Based on Stage 1 results, experts recommended scales A (with slight modifications) and B be investigated further. In Stage 2, similar proportions of children logically ranked the images used in modified scales A (69.7%) and B (75.7%). However, a majority of children ranked the images in scale B in the reverse order (60.0%). Slightly more children were able to interpret the images in the context of their asthma using scale B (57.6%) than modified scale A (48.5%). Children and parents/caregivers preferred modified scale A over scale B (78.8% and 90.9%, respectively). Compared with the original C-ACT, most children selected the same response option on items using both scales, supporting equivalency. Following review of Stage 2 results, all five experts agreed modified scale A was the optimal response scale.

**Conclusions:**

This study developed alternative response option images for use in the C-ACT and provides qualitative evidence of the equivalency of these response options to the originals.

**Supplementary Information:**

The online version contains supplementary material available at 10.1186/s41687-021-00390-2.

## Plain English summary

Accurate monitoring of the symptoms associated with pediatric asthma is important when managing the condition. The CHILDHOOD ASTHMA CONTROL TEST (C-ACT) is a questionnaire widely used to measure asthma severity in young children (aged 4–11 years). Each question answered by the child in the C-ACT has four possible answer choices. To help children answer, each choice is presented alongside an image of a male child’s face ranging from sad to happy. However, there are concerns that the images used are not culturally universal and could be misinterpreted—due to difficulties translating to electronic formats and a lack of differentiation between the images used. Through interviewing children with asthma, we aimed to address these concerns by developing and testing new images. Alternative image options developed included simpler faces, circles of decreasing size and squares of decreasing quantity. Children aged 4–11 years old were interviewed to test whether they understood the response scale using the new images and if they answered in the same way as with the original images. Interviews were conducted in two stages, with expert guidance at key stages. Results showed that children can interpret and understand the newly developed images and that they answer the questions the same as they would using the original images. These new images have the advantages of being culturally neutral and easier to implement on an electronic device.

**Supplementary Information:**

The online version contains supplementary material available at 10.1186/s41687-021-00390-2.

## Background

Asthma affects as many as 339 million people worldwide [1], with a global wheezing prevalence rate of up to 8.6% [2]. Asthma commonly presents during childhood and is the most common chronic condition among children [3, 4]. Asthma symptoms, which include wheezing, chest tightness, shortness of breath and cough, typically vary in frequency and intensity [1] and contribute to the burden—felt by both the family and the child—associated with the condition [5]. As poor symptom control is often associated with increased risk of asthma exacerbations, one of the main goals of asthma management is to achieve and maintain good symptom control [5, 6]. To reach this goal, it is essential to monitor asthma symptoms in children, in order to obtain and maintain control of the condition and provide an opportunity to relieve asthma-related impairment as much as possible [7].

The CHILDHOOD ASTHMA CONTROL TEST (C-ACT) is a validated patient- and caregiver-reported outcome instrument used to evaluate asthma severity in children aged 4–11 years [8–10]. The C-ACT was originally developed in 2007 by GlaxoSmithKline plc. (GSK). It is a widely utilized tool, often used as an endpoint in pediatric asthma trials and recommended in clinical guidelines as being of value when assessing asthma control in general clinical practice [11]. The C-ACT has shown good diagnostic accuracy for identifying both controlled and uncontrolled asthma and is commonly used in combination with factors such as school absence and limitations of daily activities to identify uncontrolled asthma [12]. The C-ACT therefore provides a standardized and validated instrument for assessing asthma control. The instrument includes four items that are completed by the child and three items that are completed by a parent or caregiver [8–10] (Additional file 1: Figure S1). The items completed by the child are answered on a 0–3 response scale, whereas the items completed by the parent are answered on a 0–5 response scale. For the child-reported items, each response level includes a verbal descriptor accompanied by one of four faces that range from sad to happy in expression, in order to assist the child in their interpretation (Additional file 1: Figure S1). The C-ACT can be completed on paper or electronically, and a web-based version is freely available (accessible via desktop computer, laptop, tablet or smartphone) [13, 14].

Despite its widespread use and proven clinical utility [15], GSK have concerns that the current response option images used in the C-ACT (black and white illustrations of a male child’s face in varying degrees of happiness/unhappiness) are not culturally universal (Fig. 1.1). Cultural universality is important as the C-ACT is widely used globally, and it is therefore necessary to ensure that there is measurement equivalence and acceptability of the measure by children across different countries and languages. Another concern is that the images included may be too detailed to be differentiated on small electronic interfaces such as smartphones, particularly for young children. Also, young children may have very literal interpretations and not endorse a crying face (indicating worst symptom level) if they, personally, do not cry in response to asthma experiences [16, 17]. The images themselves might also be too complex and the differences between them too subtle, making it difficult for children to interpret and distinguish one from another. It is also worth considering that there is likely to be increased uptake of technology-based assessments in the future; therefore, improving any interpretation issues associated with electronic interfaces will be crucial. Given these concerns, this study sought to identify, evaluate and select an alternative set of images to support the response scale for the child-completed C-ACT items.

**Fig. 1:**
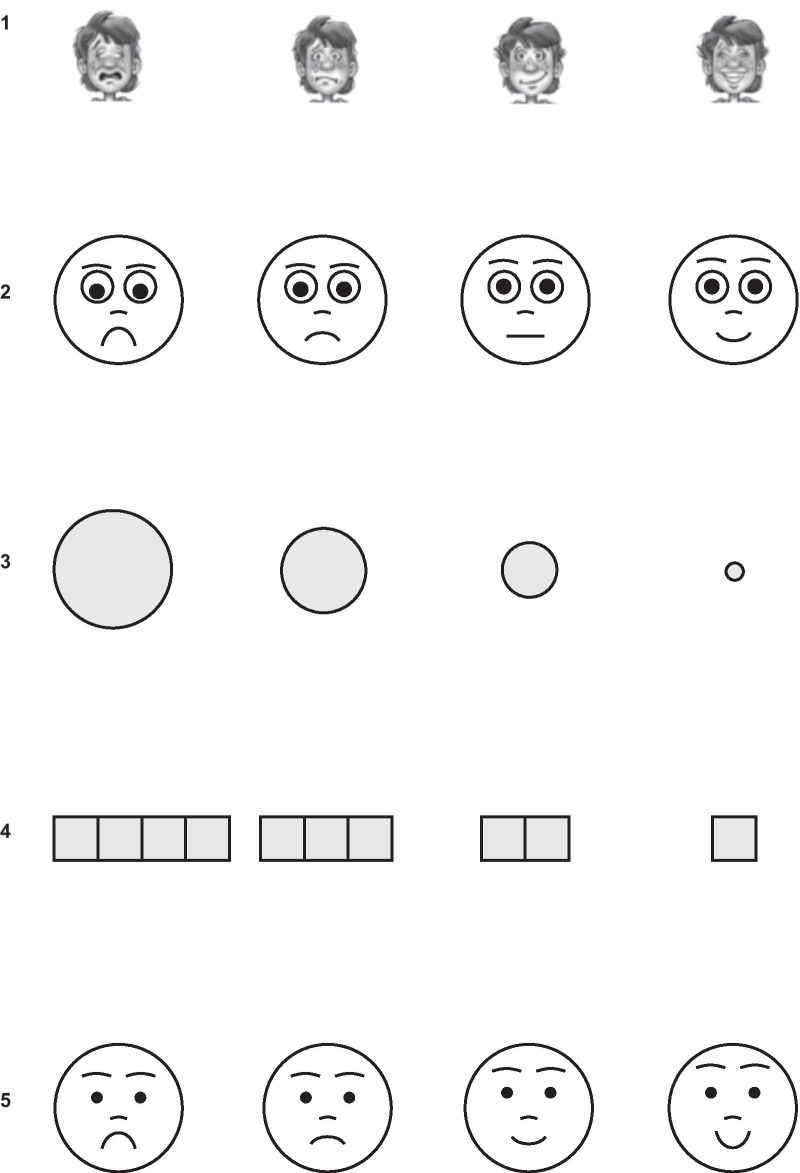
Response options. **1** Original C-ACT response options; **2** Scale A (simple faces); **3** Scale B (circles of decreasing size); **4** Scale C (squares of decreasing quantity); **5** Modified Scale A for Stage 2 (simplified faces with modified eyes and mouth), developed after Stage 1 analysis; response options from left to right: option 1 (negative response), option 2 (slight negative response), option 3 (slight positive response), option 4 (positive response). C-ACT, CHILDHOOD ASTHMA CONTROL TEST

This study aimed to assess alternative response image options for use in the C-ACT through qualitative cognitive interviews with children with asthma and their parents/caregivers, using both paper and electronic modes of administration. This was done to confirm qualitative equivalence of the images, to ensure that children can clearly differentiate between the images (ordered from bad to good asthma), and to ensure that each image represents various points along that continuum (e.g., low or high symptom burden). An additional aim was to evaluate the content validity of the electronic version of the new response option images, to ensure the images were suitable to be viewed on a small screen such as a mobile phone or tablet.

## Methods

### Study design

This was a cross-sectional, qualitative interview study with children aged 4–11 years old who had a clinical diagnosis of asthma and their parents/caregivers. The recruitment period for the study was from 12 February 2019 to 15 October 2019. Interviews were conducted across four countries: the United States of America (US), Spain, Poland and Argentina, thus representing a diverse range of cultures across North America, Europe, and Latin America. Additionally, five experts with relevant expertise from a variety of fields (including asthma, measurement and pediatrics) were identified, briefed on the project objectives, and invited to be members of an expert panel—the role of which was to provide clinical and methodological input at key stages throughout the study. Ethical approval in the US was provided by the Copernicus Group institutional review board (IRB), a centralized IRB. Additional ethical approval was not a requirement in Poland, but parents/caregivers had to provide written informed consent for their child’s inclusion in the study. Written informed consent was obtained from a parent/guardian for all children interviewed prior to interview and any study activities. Written assent was also obtained from children aged ≥ 10 years. Verbal consent was obtained from all children at the start of the interview. In Argentina, documents approved by the Copernicus Group IRB were provided to the site to achieve local site approval. For Spain, the Surveillance Unit of FarmaIndustria reviewed and approved the study.

The objectives of the study were to: (1) develop new response option images for the child-completed items (1–4) of the C-ACT scale; and (2) evaluate content validity and qualitative equivalency of these alternative images versus the original C-ACT images. The original C-ACT images were used as the standard for equivalence testing due to widespread utilization of the C-ACT as a tool in pediatric asthma trials and guidelines. It has been proven to have good psychometric measurement properties and the scale itself is robust; hence, we did not want to change the response options or scores, we simply wanted to ensure that the faces mean the same in terms of response to patients. Therefore, we aimed to retain the value of the original scale but make the faces more relevant, appropriate and culturally inclusive. Qualitative equivalency was defined as the comparability of the appropriateness of the response scales as a measurement tool across various geographical or cultural settings [18]. It was intended that the alternative response option images should be recognizable and consistently understood by children, support qualitative version equivalence (number and wording/order of the response options should remain constant), and be amenable to easy reproduction across a range of interfaces. To address the objectives, qualitative cognitive interviews were conducted with children aged 4–11 years. The interviews aimed to: determine the most appropriate images for the response scale based on acceptability and participant understanding/interpretation of the images; explore children’s abilities to consistently understand the response option images and the accompanying verbal/numerical descriptors, on both paper and an electronic mode of administration; and assess the qualitative equivalency of the alternative response option images with the original C-ACT images. The interview guides used can be found in Additional file 1: Files 1 and 2.

A literature review of existing pediatric patient-reported outcomes (PROs), combined with input from the expert panel, informed the selection and development of three new response option image scales to be debriefed with children during the interviews (Additional file 1: Table S1). The literature review helped to identify possible alternative images that could be used to support the C-ACT response scale and to investigate the value of incorporating image-based response options. Based on the literature review, three alternative response option images were developed for comparative analysis via qualitative evaluation. As the existing C-ACT uses faces in the response option images, and given the widespread use of faces in other pediatric PRO response scales (22/27; Additional file 1: Table S1), these options included one face-based response scale. The alternative response option images developed included: scale A (simple **faces**), scale B (**circles** of decreasing size), and scale C (**squares** of decreasing quantity) (Fig. 1.2–.4).

Interviews were conducted in two stages. Stage 1 interviews compared three potential alternative versions of the response option images for the child-completed items (1–4) of the C-ACT. Children were split into three groups, with two of the three modified response option image scales tested in each group. All three sets of response option images were not debriefed with every child as it was felt that children would find the task of debriefing and then comparing three scales in one interview too demanding—possibly impacting on the quality of the data. Stage 2 interviews compared the two most optimal alternative response option images identified in Stage 1 with the original C-ACT images, using an independent sample of children. Qualitative equivalence of one of the two alternative response option images versus the original C-ACT response scale was also explored in Stage 2.

**Fig. 2:**
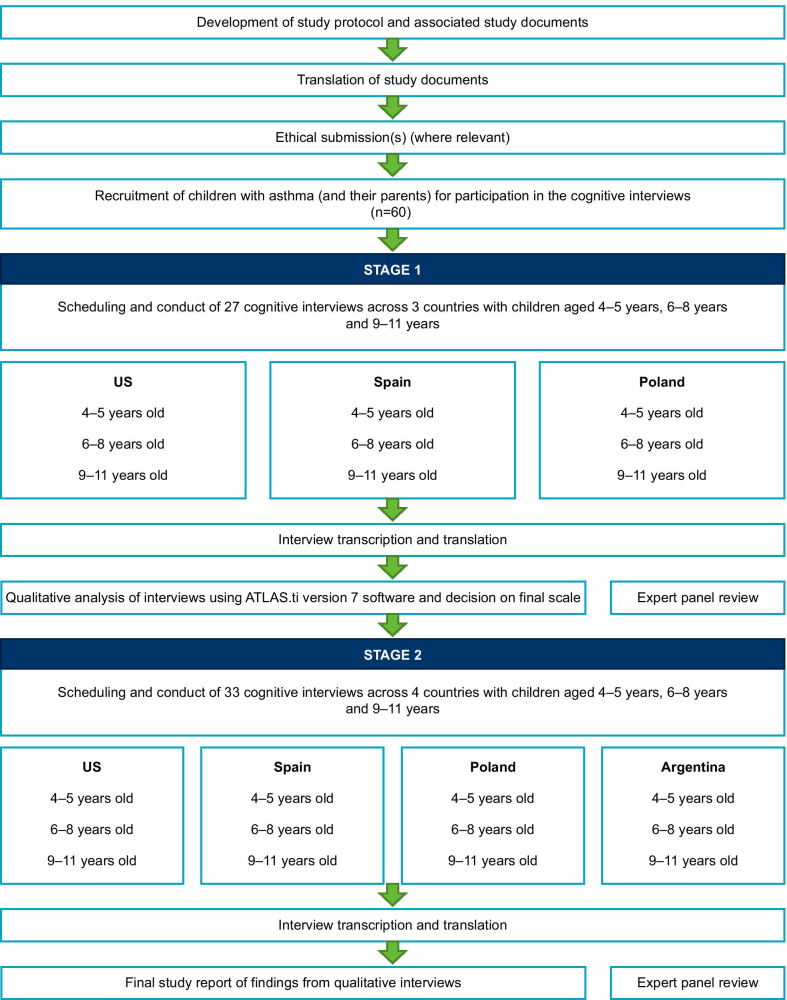
Overview of methodology

For both stages, children completed items 1–4 on the C-ACT, and their parent/caregiver completed items 5–7 following the interview. As well as answering these questions, parents/caregivers also completed a short questionnaire on the suitability of the modified images at the end of Stage 2 only. Results from each stage were presented to the expert panel for review and input. An overview of the methodology is shown in Fig. 2**.**

The response scales with the new images were developed into an electronic PRO (ePRO) format by an ePRO vendor (Kayentis). Screenshots were reviewed and approved by the project team. All the modified response options, as well as the original C-ACT, were administered to children on the ePRO during the interview, while the ranking task was performed using paper print outs. Children took part in 30-min, semi-structured, face-to-face cognitive interviews.

### Interview conduct

All interviews were conducted in the native language of the participant by Adelphi Values trained experienced qualitative interviewers, using a semi-structured interview guide. Interviews were generally 30 min in duration and took place at a central location in each country, at a prescheduled time. The same process was followed for both stages, with each interview performed by a single interviewer. Children were split into groups, with the order of scales counterbalanced within each, meaning the scales were distributed evenly throughout the age groups and countries. Children used paper copies of the response scales to arrange the images in rank order (from one = ‘bad asthma’ to four = ‘good asthma’). This allowed assessment of whether children could place images in a logical order on a scale from bad asthma symptoms to good asthma symptoms (e.g., selecting the smallest circle to represent ‘good asthma’ and the largest circle for ‘bad asthma’ for scale C). The context of understanding was also reported; for example, whether children understood the images within the context of their asthma or the emotions of the face scales. Following this ranking process, children completed C-ACT items 1–4 using the modified images on an electronic format. This allowed assessment of usability and appropriateness of the new response option images in an electronic format alongside the testing of content validity.

**Fig. 3:**
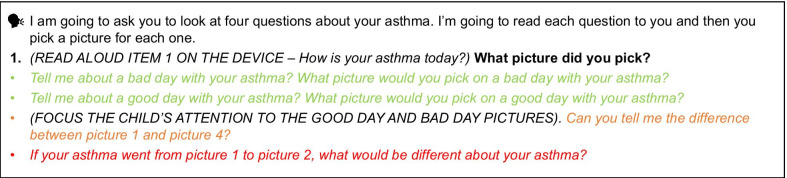
Example of traffic light system questioning from the interview guide

The interviews were conducted using a ‘think aloud’ process in the presence of the parent/caregiver. The participant was presented with a version of the scales and asked to speak their thoughts aloud as they completed each item. The interview questions were labelled in the interview guide (not seen by the children) (Fig. 3) using a traffic light system; questions were designated as green, amber or red depending on how difficult they were to understand. Green questions were asked to all children and informed the fundamental research questions regarding understanding and appropriateness of the response options. The follow up amber questions were slightly more challenging and were asked if the interviewer deemed that the child had understood the previous green probe. Red questions were only asked to those with higher cognitive abilities, who had understood both the green and amber probes. This approach ensured a degree of standardization, with fundamental questions (i.e., green questions, which were key to the research objectives) being asked to all children, while also allowing each interview to be tailored to an individual child’s ability. Cognitive debriefing questions explored the appropriateness, acceptability and understanding of the response option images for each of the four items. All interviews were audio-recorded, transcribed verbatim and translated (into English) as relevant and anonymized. Transcripts were translated by ‘TransPerfect’, who are certified to the ISO 9001 and ISO 17100 standards and ensure all transcripts are assessed for quality assurance. They also provide detailed training to their translation team.

### Study population

The study targeted a sample size of 60 children who were recruited by primary care physicians, general practitioners or respiratory specialists: 20 aged 4–5 years, 20 aged 6–8 years and 20 aged 9–11 years. The target sample was chosen in line with recommendations from the ISPOR (The Professional Society for Health Economics and Outcomes Research) Good Research Practices Task Force report [19]. Target sample sizes were established for country, age, gender and medication step (Additional file 1: Table S2). Medication steps—assessed to highlight potential differences between countries—were based on medication prescription as described in Additional file 1: Table S3. A sample of 30 children was targeted for both Stage 1 and Stage 2 interviews.

To be included in the study, children had to be aged between 4 and 11 years with a physician-confirmed diagnosis at least 6 months prior to this study, as defined by national and international asthma guidelines (e.g., National Heart, Lung, and Blood Institute Expert Panel Report 3, 2012) [20]. Patients were excluded if they had no physician-confirmed diagnosis of asthma or if they had been diagnosed with a co-morbid chronic respiratory condition. Children were also excluded if they had a history of cognitive or developmental delay that could have affected their ability to participate in the interview. Children who met the study inclusion/exclusion criteria were identified by their physician and invited to attend the physician’s office.

### Data analysis

Qualitative, thematic analysis of the anonymized transcripts was conducted using ATLAS.ti version 7 software [21]. Stage 1 interview transcripts were analyzed to determine participant understanding and perceived appropriateness of the response image options, and to determine which images children preferred and the reasoning for this. Based on the findings from Stage 1, two of the alternative response scales were chosen and taken forward for cognitive testing in Stage 2 of the qualitative interviews. During Stage 2, analysis of the interview transcripts assessed children’s understanding and the appropriateness of the images. In addition, equivalence codes were included to determine the conceptual equivalence of the updated response scale images with the original C-ACT images. Equivalence was assessed qualitatively for each item and was deemed to have been met if children selected the same response for the alternative scale and the C-ACT scale.

## Results

### Demographic and clinical characteristics

In total, 60 children were recruited into the study. In Stage 1, 27 children from the US, Spain, and Poland were split into three groups, with each group debriefed on two of the three image scales. Due to timelines associated with recruitment and ethics, no Argentinian children were involved in Stage 1. Originally, 18 children were to be debriefed for each scale; however, slightly more children were debriefed on scale B compared to scale C. In Stage 2, a total of 33 children from the US, Spain, Poland, and Argentina were debriefed on both scale A and scale B. Demographics and clinical characteristics from Stage 1 and Stage 2 are presented in Table 1.

**Table 1:** Demographic and baseline characteristics of children for Stage 1 and Stage 2

Demographics Stage 1	US (*n* = 9)	Spain (*n* = 9)	Poland (*n* = 9)	Total (*n* = 27)
Age				
Mean (min, max)	7.0 (4, 11)	7.6 (4, 11)	7.3 (4, 11)	7.3 (4, 11)
Gender, *n* (%)				
Male	4 (44.4)	6 (66.7)	6 (66.7)	16 (59.3)
Female	5 (55.6)	3 (33.3)	3 (33.3)	11 (40.7)
Age at diagnosis, *n* (%)				
≤ 5 years	9 (100.0)	6 (66.7)	7 (77.8)	22 (81.5)
≥ 6 to ≤ 10 years	–	3 (33.3)	2 (22.2)	5 (18.5)
Ethnicity, *n* (%)				
Hispanic or Latino	1 (11.1)	9 (100.0)	–	10 (37.0)
Non-Hispanic or Latino	8 (88.9)	–	9 (100.0)	17 (63.0)
Race, *n* (%)				
Caucasian	3 (33.3)	8 (88.9)	9 (100.0)	20 (74.1)
Black/African American	6 (66.7)	–	–	6 (22.2)
Asian	–	–	–	–
Multiracial	–	1 (11.1)	–	1 (3.7)
Asthma presentation & treatment, *n* (%)				
Step 1	2 (22.2)	–	–	2 (7.4)
Step 2	2 (22.2)	2 (22.2)	7 (77.8)	11 (40.7)
Step 3	2 (22.2)	4 (44.4)	2 (22.2)	8 (29.6)
Step 4	3 (33.3)	2 (22.2)	–	5 (18.5)
Step 5	–	1 (11.1)	–	1 (3.7)

Demographics Stage 2	US (*n* = 6)	Spain (*n* = 6)	Poland (*n* = 6)	Argentina (*n* = 15)	Total (*n* = 33)
Age					
Mean (min, max)	8.2 (4, 11)	7.3 (5, 11)	7.6 (4, 11)	7.2 (4, 11)	7.5 (4, 11)
Gender, *n* (%)					
Male	3 (50.0)	3 (50.0)	3 (50.0)	8 (53.3)	17 (51.5)
Female	3 (50.0)	3 (50.0)	3 (50.0)	7 (46.7)	16 (48.5)
Age at diagnosis, *n* (%)					
≤ 5 years	5 (83.3)	5 (83.3)	3 (50.0)	12 (80.0)	25 (75.8)
≥ 6 to ≤ 10 years	1 (16.7)	1 (16.7)	3 (50.0)	3 (20.0)	8 (24.2)
Ethnicity, *n* (%)					
Hispanic or Latino	2 (33.3)	5 (83.3)	–	15 (100.0)	22 (66.7)
Non-Hispanic or Latino	4 (66.7)	1 (16.7)	6 (100.0)	–	11 (33.3)
Race, *n* (%)					
Caucasian	4 (66.7)	4 (66.7)	6 (100.0)	4 (26.7)	18 (54.5)
Native American	–	–	–	10 (66.7)	10 (30.3)
Multiracial	–	2 (33.3)	–	1 (6.7)	3 (9.1)
Hispanic	1 (16.7)	–	–	–	1 (3.0)
Asian	1 (16.7)	–	–	–	1 (3.0)
Asthma presentation & treatment, *n* (%)					
Step 1	4 (66.7)	2 (33.3)	–	–	6 (18.2)
Step 2	3 (50.0)	3 (50.0)	2 (33.3)	1 (6.7)	9 (27.3)
Step 3	2 (33.3)	2 (33.3)	4 (66.7)	3 (20.0)	11 (33.3)
Step 4	-	1 (16.7)	1 (17.0)	11 (73.3)	13 (39.4)

US, United States of America

Overall, the sampling quotas were effective in ensuring demographic diversity and there was a good representation of children of different ages and both genders across the participant groups and most pre-specified recruitment quotas were met (Additional file 1: Table S4), ensuring a diverse sample from different geographical regions. The average age of children was similar in Stages 1 and 2, with ages of 7.3 years (range 4–11 years) and 7.5 years (range 4–11 years), respectively. Sex was equally represented in Stage 2 (female, 48.5%; male, 51.5%), whereas in Stage 1 there were more males than females (59.3% vs 40.7%). In terms of ethnicity, the majority of children were non-Hispanic or Latino (63.0%) in Stage 1, while the reverse was true in Stage 2, where the majority of children were Hispanic or Latino (66.7%). Caucasian was the most represented race in both stages (Stage 1, 74.1%; Stage 2, 54.5%). Country representation was split equally in each stage.

### Stage 1 results

#### Scale A (simple faces)

A majority of children were able to rank the response option images in scale A in a logical order (Fig. 4.1) (*n* = 12/18; 66.7%). Approximately half of the children (*n* = 10/18; 55.6%) demonstrated the ability to interpret the images within the context of their asthma, and a smaller percentage (*n* = 7/18; 38.8%) referred to their emotions when interpreting the images (Fig. 4.2). Seven children (*n* = 7/18; 38.8%) selected inappropriate image options to represent a good day and bad day with their asthma for at least one of the four C-ACT items. Inappropriate options were more frequently selected for responses representing a bad day (e.g., smiling face representing a bad day) than for those representing a good day (e.g., sad face representing a good day). Three of the children who selected inappropriate options were aged 4–5 years, two were aged 6–8 years, and two were aged 11 years. Scale A response option images were liked by the majority of children (*n* = 12/18; 66.7%) and were the images most frequently preferred by children when compared with the other alternative response option images (Fig. 5). Scale A was selected as the preferred scale by all children who compared scale A with scale C and by half of the children who compared scale A with scale B (*n* = 5/10; 50.0%).

**Fig. 4:**
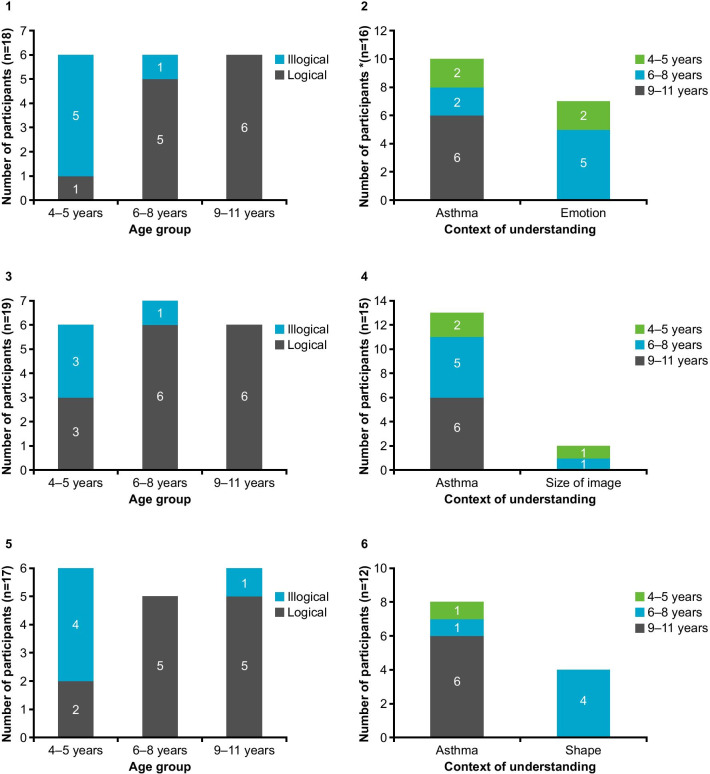
Stage 1 logical and illogical ranking and context of understanding. **1** Scale A (logical and illogical ranking); **2** Scale A (context of understanding); **3** Scale B (logical and illogical ranking); **4** Scale B (context of understanding); **5** Scale C (logical and illogical ranking); **6** Scale C (context of understanding). Logical and illogical ranking: represents the number of patients who ranked the options in a logical sequence. Context of understanding: for scale A conveyed understanding both in the context of asthma and the emotions portrayed by the image; for scale B conveyed their understanding in the context of their asthma and the size of the images; for scale C conveyed understanding both in the context of asthma and the shape of the images

**Fig. 5:**
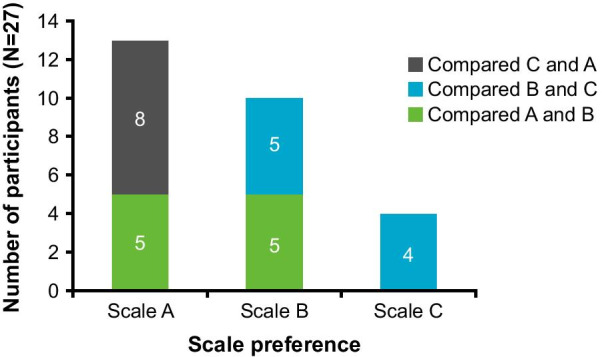
Stage 1 participant preference for scale A (simple faces), scale B (circles of decreasing size), or scale C (squares of decreasing quantity). Each bar illustrates the number of children who preferred the scale denoted, whilst the color represents the scale it was being compared with

#### Scale B (circles of decreasing size)

More children were able to rank the response option images in a logical order with scale B (*n* = 15/19; 79.0%) than with scale A and scale C (Fig. 4.3). However, four of these children (*n* = 4/15; 26.7%) ordered the images in the reverse order, selecting the smallest circle to represent ‘bad asthma’ and the largest circle for ‘good asthma’. More children were also able to interpret the images within the context of their asthma (*n* = 13/15; 86.7%) compared to scale A and scale C (Fig. 4.4). Eight out of 19 children (42.1%) selected inappropriate options to represent a good day and bad day with their asthma, more commonly for a bad day, for at least one of the four C-ACT items. Scale B response option images were liked by the majority of children (*n* = 13/19; 68.4%); however, two children suggested reordering the direction of the scale (*n* = 2/19; 10.5%). Scale B was the second most preferred scale by children (Fig. 5)—selected by ten children in total -when compared with both scale A (*n* = 5/10; 50.0%) and scale C (*n* = 5/9; 55.5%).

#### Scale C (squares of decreasing quantity)

Most children (*n* = 12/17; 70.6%) were able to rank the response option images in scale C in a logical order (Fig. 4.5); however, over half of these children (*n* = 7/12; 58.3%) ordered the images in the reverse order to that of the modified scale, selecting the single square to represent ‘bad asthma’ and four squares for ‘good asthma’. Fewer children were able to interpret the images within the context of their asthma (*n* = 8/17; 47.5%) compared to scale A and scale B (Fig. 4.6), and this was more prevalent among older children aged 9–11 years (*n* = 6/8; 75.0%). Eight out of 17 children (47.1%) selected inappropriate options to represent a good day and bad day with their asthma for at least one of the four C-ACT items, more commonly for a bad day; two of these children were aged 4–5 years, five were aged 6–8 years, and one was aged 10 years. Scale C response option images were liked by the majority of children (*n* = 11/17; 64.7%); however, scale C was the least preferred scale by children when compared with the other alternative response option images (Fig. 5).

### Stage 1 expert panel review

Following the completion of Stage 1 interviews, the expert panel convened to review the findings. Based on the comparable performance of scale A and scale B, it was recommended that both scales should be further investigated in Stage 2 interviews. Following feedback from children, the expert panel, and the study research team, scale A images (Fig. 1.2) were modified slightly to make the faces appear more friendly and easier to distinguish (Fig. 1.5); scale B response option images were left unchanged.

### Stage 2 results

#### Modified scale A (simple faces)

Most children could rank the response option images in a logical order with modified scale A (*n* = 23/33; 69.7%) (Fig. 6.1). The majority of children demonstrated an overall understanding of the differences between the four response option images in scale A (*n* = 23/33; 69.7%), most of whom interpreted the images in the context of asthma (*n* = 16/23, 69.6%), with a smaller number interpreting the emotions (*n* = 13/23, 56.5%) (Fig. 6.2). Eight children (*n* = 8/33; 24.2%) selected inappropriate options to represent a good day and bad day with their asthma. Five children were aged 4–5 years, two children were aged 6–8 years, and one was aged 11 years. Modified scale A response option images were liked by the majority of children (*n* = 23/33; 69.7%) and this was the preferred scale by children (*n* = 26/33; 78.8%) and parents/caregivers (*n* = 30/33; 90.9%) (Figs. 7.1, .2). Children also preferred modified scale A images to the original C-ACT scale images (*n* = 10/15; 66.7%) (Fig. 7.3).

**Fig. 6:**
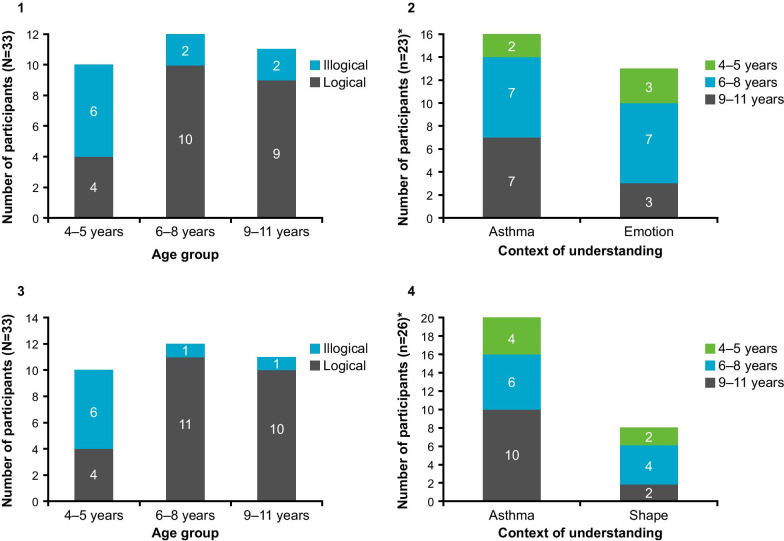
Stage 2 logical and illogical ranking, scale understanding, and context of understanding. **1** Modified scale A (logical and illogical ranking); **2** Modified scale A (context of understanding); **3** Scale B (logical and illogical ranking); **4** Scale B (context of understanding). Logical and illogical ranking: represents the number of children who ranked the options in a logical sequence. Context of understanding: for modified scale A conveyed understanding both in the context of asthma and the emotions portrayed by the image; for scale B conveyed their understanding in the context of their asthma and the shape of the images. *Children conveyed understanding both in the context of asthma and emotions (six and two in 6.2 and 6.4, respectively)

**Fig. 7:**
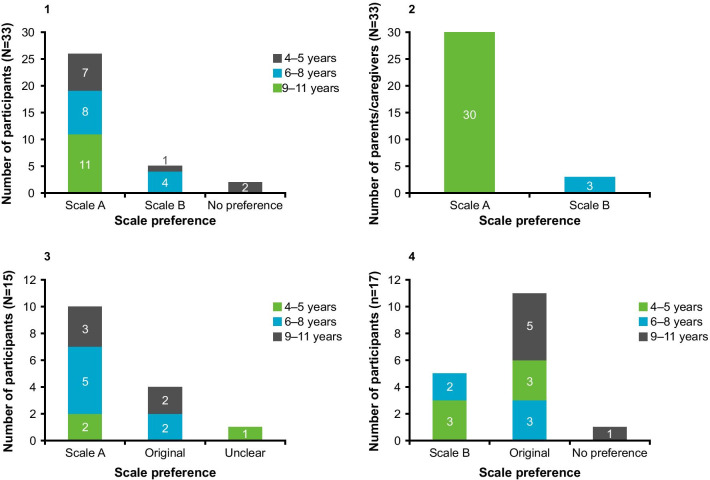
Comparison of scales. **1** Participant preference (modified scale A vs scale B); **2** Parent/caregiver preference (modified scale A vs scale B); **3** Participant preference (modified scale A vs original C-ACT); **4** Participant preference (scale B vs original C-ACT). C-ACT, CHILDHOOD ASTHMA CONTROL TEST

#### Scale B (circles of decreasing size)

A similar proportion of children could rank the response option images in a logical order (*n* = 25/33; 75.7%) compared with modified scale A (*n* = 23/33; 69.7%) (Fig. 6.3). However, most children (*n* = 15/25; 60.0%) ordered the images in the reverse order to that of the modified scale, selecting the smallest circle to represent ‘bad asthma’ and the largest circle for ‘good asthma’. The majority of children demonstrated an overall understanding of the differences between the four response option images in scale A (*n* = 26/33; 78.8%), most of whom interpreted the images in the context of asthma (*n* = 20/26, 77.9%) (Fig. 6.4). Using scale B, nine children (*n* = 9/33; 27.3%) selected inappropriate options to represent a good day and bad day with their asthma for at least one of the four C-ACT items. Five children were aged 4–5 years, three children were aged 6–8 years, and one was aged 10 years. Scale B response option images were liked by the majority of children (*n* = 23/33; 69.7%) but were preferred less than the images used in both modified scale A (*n* = 5/33; 15.2%) and the original C-ACT (*n* = 5/17; 29.4%) by children (Figs. 7.1, .4) and parents/caregivers (*n* = 3/30; 10%) (Fig. 7.2).

#### Equivalency of responses with the original C-ACT response option scale

The majority of children selected the same response option images across all four scale items when answering with either modified scale A (*n* = 39/64; 60.9%) or scale B (*n* = 48/68; 70.6%), compared with the original C-ACT scale, supporting the equivalency of both the modified scale A and scale B response option images with the original C-ACT response option scale (Table 2).

**Table 2:** Equivalency of combined responses across C-ACT items 1–4, for the comparison of response option image selection for Stage 2 scales, (A) modified scale A (simple faces), and (B) scale B (circles of decreasing size) with the original C-ACT

(A)	Original C-ACT			
**Scale A**	Response option 0 (negative response)	Response option 1 (slight negative response)	Response option 2 (slight positive response)	Response option 3 (positive response)
Response option 0 (negative response)	4^*^	1	-	-
Response option 1 (slight negative response)	2	4^*^	2	2
Response option 2 (slight positive response)	4	3	17^*^	3
Response option 3 (positive response)	3	-	5	14^*^

(B)	Original C-ACT			
**Scale B**	Response option 0 (negative response)	Response option 1 (slight negative response)	Response option 2 (slight positive response)	Response option 3 (positive response)
Response option 0 (negative response)	3*	1	1	6
Response option 1 (slight negative response)	1	2*	3	1
Response option 2 (slight positive response)	1	1	17*	1
Response option 3 (positive response)	1	1	2	26*

C-ACT, CHILDHOOD ASTHMA CONTROL TEST

*Values in bold represent the same response options images being selected in both scales

In total, 11 children selected different response option images using scale A compared with the original C-ACT response scale, across all four C-ACT items (n = 11/16; 68.8%). Of these, eight children repeatedly selected different response options for two or more C-ACT items. These participants were majority male (n = 5/8; 62.5%) but no age range trends were identified. Similarly, 10 participants selected different response option images using scale B compared to the original C-ACT response scale, across all four C-ACT items (n = 10/17; 58.8%). Of these, five participants repeatedly selected different response options in two or more C-ACT items (n = 5/17; 29.4%). These participants were all male, and three out of five of them were aged 4–5 years.

### Stage 2 expert panel review

Following the completion of Stage 2 cognitive debriefing interviews, the expert panel reconvened to examine the findings of the two modified response scales tested; all five experts who provided feedback reported a preference for modified scale A and selected these as the response option images to be used within a modified C-ACT scale. The rationale for this preference from the four experts included: children demonstrating a good level of understanding of the scale (*n* = 2); the images displaying emotions related to asthma aiding participant understanding (*n* = 2); children showing a preference for the images (Expert 2). The images addressed the concerns regarding the generalizability of the original C-ACT response option images (Expert 4), while remaining more similar to the original scale compared to the other two modified scales (Expert 2).

## Discussion

The aim of this cross-sectional, qualitative interview study was to identify, evaluate and select a new culturally universal set of response option images for the child-completed items of the C-ACT that: support interpretation and completion of the response scale; are recognized by children as ordered in relation to each other; are considered conceptually equivalent with the existing version; and are amenable to easy reproduction across a range of interfaces. In Stage 1, three scales were evaluated and based on the results and expert feedback, scale A (which was modified) and scale B were both selected for further study in Stage 2.

Both scale A (simple faces) and scale B (circles of decreasing size) response option images demonstrated strengths as alternative response option images for the four C-ACT items. The response option images in both scales were ranked in a logical order by the majority of children, irrespective of age group or country. However, for scale B, a number of children ranked the response option images in the reverse order, selecting the smallest circle to represent ‘bad asthma’ and the largest circle for ‘good asthma’ rather than perceiving the circle size to represent an increase in asthma experience of the item attribute, highlighting the potential issue of bidirectionality in the interpretation of the images with scale B. This reinforces findings from previous studies, in which the relatability of shape-based scales has been called into question within pediatric populations [22–25]. Children were able to interpret the response option images of both scales in the context of their asthma, with numerically more able to do this using scale B. In the present study, there was a concern that children could focus on the emotional component when interpreting the faces in scale A and interpret these literally rather than as a representation of their asthma—an issue that has been recognized in similar scales relating to pain [26]. However, the findings show that while some children did interpret the response options in the context of emotions, the findings suggested that this aided appropriate interpretation, and even more children conveyed understanding in the context of their asthma. It is also worth noting that, when asked, children preferred using the faces compared with other response image options. The majority of children were able to select appropriate response option images to represent a good day and bad day with their asthma for both scale A and scale B, with more variation being noted for bad day selection. Overall, more children selected inappropriate response option images when using scale B compared to scale A. In general, unsurprisingly, children aged 4–5 years demonstrated a lower level of understanding on both modified response scales, compared to older children aged 6–11 years. During the assessment of qualitative equivalence with the original C-ACT response scale, the majority of children selected the same response option across all four items whether using either scale A or scale B versus the original C-ACT. Equivalence was slightly better between scale B and the original C-ACT, with fewer children repeatedly selecting different response options across items. Younger children selected different options on the repeat measure; however, it should be noted that when children of a young age are repeatedly asked the same or similar questions, as was the case for the equivalency testing, it is not uncommon for children to change the responses they provide [27, 28]. In some cases, the child may assume something was wrong with their first answer, influencing the findings on the apparent understanding and equivalency of the modified scales—something which occurred more often in scale A than scale B.

Data from this study provide support for the qualitative equivalency of the alternative response scales proposed and suggest that children across all ages and cultures tested were understanding and interpreting the images in a way that is consistent with the original C-ACT images. Data collected from the parent/caregiver questionnaire further supported participant preference for scale A. The majority (90.9%) of the parents/caregivers of children, irrespective of country, race or ethnicity, reported that their child could evaluate their symptoms with the simple face images included in modified scale A, suggesting that these updated simple face images are appropriately neutral, relatable and suitable for use cross-culturally.

### Limitations

This was a qualitative study to support an update of the response option images and provide initial qualitative evidence regarding the equivalence with the existing images. The sample size was appropriate for such qualitative exploration. However, further research to confirm the equivalency of the newly developed response option images with those in the original C-ACT scale, within a larger sample size, that would support statistical analysis of the level of equivalence is recommended. Further quantitative research should assess the equivalency of the newly developed response option images with those in the original C-ACT scale using a larger sample size and quantitative methods for evaluating psychometric equivalency. The use of a larger sample size would also allow for quantitative statistical analysis of the reliability and validity of the measures. Whilst the traffic light system used for tailoring questions to each child’s ability offered a means of standardization, it is possible that it could have introduced bias in the selection of images—in asking certain questions of those of higher cognitive function, we cannot exclude the possibility that data may favor more highly performing (possibly older) children. Although we attempted to standardize the interview process across all children, the level of questioning was tailored to some extent to the developmental ability of the child via the traffic light system. However, there were perhaps limits to how much this could be done while retaining adequate standardization, given the range of different developmental stages of the children. To minimize the influence of unintentional parental input, parents/caregivers were asked to sit out of the child’s eyeline and asked not to directly respond on behalf of the child; however, in some instances, particularly with very young children, the parent/caregiver provided encouragement to the child. Not all sampling quota targets were met, and children were predominantly Caucasian and non-Hispanic. Another limitation of this study was the omission of non-Western countries from the research—although simple faces are displayed in many electronic interfaces commonly used by children, in cultures with less intense use of electronics, this measure may be problematic. Therefore, further analysis in these populations would be of interest in the future to further affirm the universality of the measure. The suitability of the alternative response option images should ideally be further explored across a wider variety of cultures, races and ethnicities.

## Conclusions

The present study assessed alternative image options for use in the C-ACT through qualitative cognitive interviews with children. Its findings provide qualitative evidence of the equivalency of the response scales. Findings were comparable for modified scale A and scale B; however, the potential disconnection between the direction of the shapes and the concepts assessed on scale B may compromise the consistent and accurate use of this scale with pediatric populations. The faces used in modified scale A adopted a simple design that was clearly understood by children across all age groups and was preferred by parents/caregivers. The emotions associated with the faces also supported understanding and interpretation of modified scale A, possibly aiding self-reports of asthma severity within young populations. The modified (and more simplistic) image-based response options could improve robustness of the C-ACT, through improving understanding across diverse populations and improving its migration to electronic devices. To explore the suitability of these modified response option images further, additional quantitative equivalence testing with the original C-ACT is recommended.

## Supplementary Information

**Additional file 1.** Supplementary material.

## Data Availability

Information on GlaxoSmithKline plc.’s data sharing commitments and requesting access to anonymized individual participant data and associated documents can be found at www.clinicalstudydatarequest.com.

## Abbreviations

C-ACT: CHILDHOOD ASTHMA CONTROL TEST
ePRO: Electronic patient-reported outcome
GSK: GlaxoSmithKline plc.
IRB: Institutional review board
PRO: Patient-reported outcome
US: United States of America
