# Supplementary material for: Development and equivalence of new faces for inclusion in the Childhood Asthma Control Test (C-ACT) response scale
Source: J Patient Rep Outcomes. 2021 Nov 6;5:118. doi: 10.1186/s41687-021-00390-2 (PMC8572277; doi:10.1186/s41687-021-00390-2)
Supplement: Supplementary file 1 — Additional file 1. Supplementary material. [file 41687_2021_390_MOESM1_ESM.docx]

**Supplementary appendix: Development and equivalence of new faces for inclusion in the Childhood Asthma Control Test (C-ACT) response scale**

**Supplementary Table S1** Summary of key findings from the literature review

| **Scale type** | | **Key considerations** |
| --- | --- | --- |
| Face scales | 22/27 PROs identified used face scales including:   - simple line drawings - cartoon characters - icons or emotions - detailed line drawings - photographs of children | - Face scales were the most widely used in existing pediatric PROs - Animated/cartoon faces were suggested to be more friendly and attractive for younger children and to improve understanding of the scale [1, 2] - However, there are concerns over whether specific face types are relatable for children of all ages, races, ethnicities and both genders [3] - Faces that are very detailed or accurate may be difficult for younger children to interpret [4] and it may be difficult for fine detail to be apparent when presented electronically |
| **Scale type** | | **Key considerations** |
|  |  | - Confounding effect of emotion as a child’s response may be more influenced by the child’s emotional state and which face reflects that emotional state best, rather than their symptom severity [5] - Literal interpretation of the emotions represented in the images could be a problem at times e.g., a crying face used for the negative end of the scale resulting in “I didn’t choose that face because I didn’t cry” [5] - Debate over the use of smiling or neutral faces for the positive end of a scale, as concerns over the appropriateness of pairing happiness with even mild levels of symptoms such as pain and the increased likelihood of overestimations of severity [5] |
|  | Animal faces | - 2/27 scales used animal faces [6, 7] - These have the advantage of being gender neutral and not culturally specific as children will not feel like they have to physically identify with an image in order to select it as a response - Risk of distraction and danger that children may select the image they find most appealing as the animals are not specific to the concept of interest being assessed |
|  | Emojis/emoticons | - 3/27 scales used black facial features and yellow faces, synonymous with emojis - These images are used as a popular form of communication and are perhaps more culturally universal than others and easily understood by a variety of populations [8] - Evidence to suggest that the computerization of a response scale helps engage younger individuals and images are appropriate for children with low levels of literacy [9] - Images are often presented in color [10], with differing levels of detail and gradation which may cause issues when printed/copied or displayed across different platforms - Images can have different interpretations cross-culturally |
| **Scale type** | | **Key considerations** |
| Shape scales | 4/27 PROs used shape scales including:   - squares of decreasing quantity - circles of decreasing size - shapes with graduated shading showing increasingly filled boxes | - All of these scales presented the shape images alongside verbal descriptors - Shapes were presented in order of size and were most often used in the assessment of frequency, with the largest (or increased amount) representing the most frequent option on the scale (e.g., always, a lot) [11–14] - Neutral and context-free images which are not confounded by emotion or being culturally specific - Use may be restricted to an assessment of frequency, with a lack of information pertaining to their suitability for the assessment of other types of concepts such as symptom severity - Concern over the relatability of the images as children may struggle to identify with them and may not be as engaged when responding |
| Other pictorial scales | 1/27 PROs used pictorial response options to represent throat closing, chest tightness and effort of increasing severity | - Images may be too detailed and complex for younger populations to understand [14] |
| **General considerations for scale development** | | |
| Use of color | | - 6/27 of the PROs identified used color response option images [2, 8–10, 14]. The majority of images were in black and white (21/27), with only 2/27 PROs using color (traffic light system of green, amber and red) to indicate different response options [2, 10] - Difficulties related to using color included the loss of detail or information when copied or printed, the challenge of consistently presenting color across different platforms and the lack of a consistent spectrum of colors used to visually represent different severities [15] |
| Simple designs | | - Regardless of the type of image used, the literature supports a simplified design, with images that are clear, unambiguous and related to the topic being assessed [4] |
| **Scale type** | | **Key considerations** |
| Directionality | | - 16/22 face scales were presented horizontally with happy or neutral faces on the left and unhappy or painful faces on the right - One study found children showed a preference for the horizontal presentation of shapes on a response scale, as this direction reflects the one followed when reading from left to right [13] |
| Verbal/numerical descriptors | | - 16/27 PROs presented the response option images alongside verbal and/or numerical descriptors on the scale - It was suggested that children are more likely to interpret the response options as intended and in relation to the specific concept of interest if paired with appropriate descriptors |
| Number of response options | | - Two articles highlighted concerns with presenting too many response options, as children are likely to have difficultly distinguishing between each option on the scale [16, 17] - A scale with four response options has been suggested as the optimum number for a scale intended for pediatric populations [18] |

PRO, patient-reported outcome

**Supplementary Table S2** Proposed target quotas for participant interviews per country (*n* = 15)

| **Participant characteristics** | | **Children** | | | **Quota total** |
| --- | --- | --- | --- | --- | --- |
|  |  | **4–5 years** | **6–8 years** | **9–11 years** |  |
| **Age** | | 5 | 5 | 5 | 15 |
| Gender | Male | ≥2 | ≥2 | ≥2 | ≥6 |
|  | Female | ≥2 | ≥2 | ≥2 | ≥6 |
| Asthma medication step* | 1–3 | ≥1 | ≥1 | ≥1 | ≥3 |
|  | 4–6 | ≥1 | ≥1 | ≥1 | ≥3 |

*Per GINA report [19]

**Supplementary Table S3** Asthma medication step based on prescription

| **Step** | **Asthma medication** |
| --- | --- |
| **1** | Short-acting β-agonist as needed |
| **2** | Low-dose inhaled corticosteroid OR cromolyn, leukotriene receptor antagonist, nedocromil or theophylline |
| **3** | Low-dose inhaled corticosteroid plus long-acting β-agonist OR medium-dose inhaled corticosteroid |
| **4** | Medium-dose inhaled corticosteroid plus long-acting β-agonist OR medium-dose inhaled corticosteroid plus either leukotriene receptor antagonist or theophylline |
| **5** | High-dose inhaled corticosteroid plus long-acting β-agonist OR high-dose inhaled corticosteroid plus long-acting β-agonist or theophylline |
| **6** | High-dose inhaled corticosteroid plus long-acting β-agonist plus oral corticosteroid OR high dose inhaled corticosteroid plus long-acting β-agonist or theophylline plus oral corticosteroid |

Asthma medication steps per GINA report [19]

**Supplementary Table S4** Achievement of sampling quotas for the total sample in each country (*n* = 15)

| **Country** | **Quota** | | **Target quota** | | | **Overall target** | **Actual sample** | | | **Overall sample** |
| --- | --- | --- | --- | --- | --- | --- | --- | --- | --- | --- |
|  |  |  | **4–5 years** | **6–8 years** | **9–11 years** |  | **4–5 years** | **6–8 years** | **9–11 years** |  |
| **US** | Age | | 5 | **5** | 5 | 15 | 5 | **4** | 6 | 15 |
|  | Gender | Male | ≥2 | **≥2** | ≥2 | ≥6 | 2 | **1** | 2 | 6 |
|  |  | Female | ≥2 | ≥2 | ≥2 | ≥6 | 3 | 3 | 3 | 9 |
|  | Medication step | 1–3 | ≥1 | ≥1 | ≥1 | ≥3 | 4 | 3 | 5 | 12 |
|  |  | 4–6 | ≥1 | ≥1 | ≥1 | ≥3 | 1 | 1 | 1 | 3 |
| **Spain** | Age | | 5 | 5 | 5 | 15 | 5 | 5 | 5 | 15 |
|  | Gender | Male | ≥2 | ≥2 | ≥2 | ≥6 | 4 | 2 | 3 | 9 |
|  |  | Female | **≥2** | ≥2 | ≥2 | ≥6 | **1** | 3 | 2 | 6 |
|  | Medication step | 1–3 | ≥1 | ≥1 | ≥1 | ≥3 | 5 | 5 | 1 | 11 |
|  |  | 4–6 | **≥1** | **≥1** | ≥1 | ≥3 | **0** | **0** | 7 | 4 |
| **Poland** | Age | | **5** | 5 | 5 | 15 | **4** | 6 | 5 | 15 |
|  | Gender | Male | ≥2 | ≥2 | ≥2 | ≥6 | 2 | 4 | 3 | 9 |
|  |  | Female | ≥2 | ≥2 | ≥2 | ≥6 | 2 | 2 | 2 | 6 |
|  | Medication step | 1–3 | ≥1 | ≥1 | ≥1 | ≥3 | 4 | 5 | 6* | 15* |
|  |  | 4–6 | **≥1** | ≥1 | **≥1** | **≥3** | **0** | 1* | **0** | **1*** |
| **Argentina** | Age | | 5 | 5 | **5** | 15 | 5 | 6 | **4** | 15 |
|  | Gender | Male | ≥2 | ≥2 | ≥2 | ≥6 | 3 | 3 | 2 | 8 |
|  |  | Female | ≥2 | ≥2 | ≥2 | ≥6 | 2 | 3 | 2 | 7 |
|  | Medication step | 1–3 | ≥1 | ≥1 | ≥1 | ≥3 | 1 | 1 | 2 | 4 |
|  |  | 4–6 | ≥1 | ≥1 | ≥1 | ≥3 | 4 | 5 | 2 | 11 |

Bold text indicates the target quota was missed; *Reported both 1–3 and 4–6 medication steps; US, United States of America

**Supplementary Fig. S1** Original Childhood Asthma Control Test


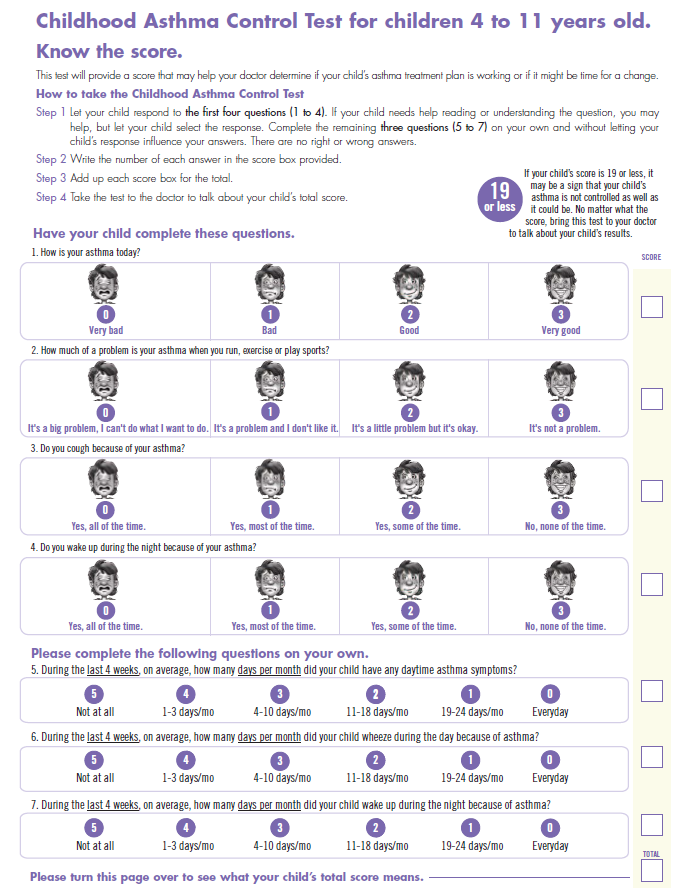


| G | K | 8 | 0 | 3 | 3 | A |  |  |  |
| --- | --- | --- | --- | --- | --- | --- | --- | --- | --- |
| Project Code | | | | | | | Subject ID | | |

**Supplementary File 1** Round 1 Interview Guide

| Date of interview: |  | / |  | / |  |
| --- | --- | --- | --- | --- | --- |
|  | Day |  | Month |  | Year |

| Location of interview: |  | , |  |
| --- | --- | --- | --- |
|  | City |  | Country |

| Name of interviewer: |  |  |  |
| --- | --- | --- | --- |
|  |  |  |  |

**Interview objectives**

The overall objective of the project is to develop and confirm the content validity and qualitative equivalency of a modified version of the C-ACT (see Appendix 1), specifically a version with updated image response options for the child-completed items (1-4). It is intended that the interviews will be conducted in two rounds. Participants will complete the modified versions of the C-ACT on an electronic hand-held device.

The specific objectives of the first round of cognitive interviews are:

- To evaluate the acceptability, relevance and understanding of up to three modified image response scales
- To assess the usability of the modified image response scales on an electronic mode of administration

The specific objective of the second round of cognitive interviews are:

- To confirm content validity and qualitative equivalency of the final modified image response scale with the original version
- To assess the usability of the modified image response scales on an electronic mode of administration

**Overview of the interview**

The interview should be conducted as follows:

1. **Introduction to the study**: Introduce Adelphi Values to the participant. Explain the objectives and the process of the interview.
2. **Consent:** Ensure written informed consent to participate has been obtained prior to the beginning of the interview. Also obtain the participant’s verbal consent for the interview to be audio-recorded.
3. **Interview:**

The interview is divided into the following sections:

- Introduction to the study and consent (5 minutes)
- Image response scale 1 (10 minutes)
  - Ranking exercise
  - Cognitive debriefing of the scale
- Image response scale 2 (10 minutes)
  - Ranking exercise
  - Cognitive debriefing of the scale
- Comparison of image response scale 1 and scale 2 (3 minutes)
- Original C-ACT (2 minutes)
  - Cognitive debriefing of the scale

In total, the interview should take approximately **30 minutes** (not including travel and scheduling time).

**Role of the interviewer and instructions**

**The role of the interviewer before the interview**

1. Check the screener to confirm that the child is eligible to take part.
2. Check the caregiver/child (only those aged >10 years old) has provided written informed consent/assent. All children must provide verbal consent for the interview to be audio recorded.
3. Check the ePRO is charged.

**Checks** **with caregivers prior to the child interview**

**Before the interview starts, briefly take the child’s parent/caregiver to the side and ask the following to aid you in probing the child**

1. **How does [the child] refer to their asthma?**

Does s/he just talk about “asthma” or “breathing problems”?

1. **How does [the child] talk about their symptoms?**

What words does s/he use to talk about breathing difficulty, breathlessness or shortness of breath?

What words do you use to talk about coughing?

**The role of the interviewer during the interview**

- **Digital Audio recording**: Ensure that the comments of the child throughout the interview are clearly recorded on the audio-recording. Audio recordings will be transcribed verbatim. Check the volume settings and positioning of the audio recorder to ensure the clarity of the audio recording. Avoid rustling papers near the recorder or jostling the recorder during the interview as this will lead to inaudible responses.
- **Interview guide:** Make a written note in the interview guide of any non-verbal communication by the patient (e.g., vocalisations or facial expressions indicating rejection or acceptance of one of your comments; apparent affect such as confusion, frustration, annoyance, etc.). Comment on the non-verbal cue and invite the patient to explain his/her feelings to ensure you have properly interpreted the gesture.
- **Manging parent/caregiver:** If it is necessary for a parent/caregiver to be present in the interview, they will be asked to sit out of the child’s eye line and asked not to directly respond on behalf the child. However, the parent/caregiver can provide encouragement to the child or explain a question to them if necessary.
- **Completion of C-ACT:** After each interview ensure the child completes items 1-4 of the original C-ACT (**on paper**) and their parent/caregiver completes items 5-7.

The role of the interviewer is to **listen** and **support:**

- Please do not give your own point of view
- Be patient and accept silence during open-ended questioning to make sure the participant has time to think about his/her response
- Help the participant stick to the topics intended in the guide
- Help the participant cross-reference and make connections that they would not do spontaneously
- Ask the participant to clarify any comments or gestures that you are not certain you understood

**Using the Interview Guide**

Throughout this guide we have coded the questions and instructions so that it is clear which questions are required and which are probes, to be used when necessary. The codes are as follows:

| UPPER CASE TEXT | **These are guidance notes for you (the interviewer)** |
| --- | --- |
| - ***Bold, italic, bulleted text*** | These are instructions to you (the interviewer) |
|  | These are statements that you read aloud word-for-word to the participant. |
| 1. **Bold text** | Questions prefixed by a specific number must be asked to all participants |
| - *Italic text* | All probes or follow up questions are bulleted – these do not necessarily need to be used, but are included to assist you in drawing further information from participants if needed. The probes vary in difficulty and follow a traffic light system, so that you (the interviewer) are able to easily identify probes which participants may find more difficult to understand. |
| - *Green italics* | If a participant understands the first question, ask this low ability follow up probe. |
| - *Amber italics* | If a participant understands the green italic probe, ask this middle ability follow up probe. |
| - *Red italics* | If a participant understands the amber italic probe, ask this high ability follow up probe. |

**Adverse Events**

**What should you do if an adverse event is mentioned?**

If during an interview, a child or patient/caregiver mentions an adverse event (AE) or product complaint related to a product of the sponsor, whether it is considered serious or not, this must be reported to the appropriate drug safety/pharmacovigilance department at the sponsor.

If the child or patient/caregiver mentions an adverse event that they or anyone else experienced while taking any GSK product the interviewer will complete an adverse event (AE) reporting form (See Appendix 2) and send to Adelphi Values via email (kate.sully@adelphivalues.com). The interviewer will complete the AE reporting form at the end of the interview that will capture information regarding the reportable event, the GSK product involved and the role of the person reporting the event. The AV trained interviewer will also seek the parent/caregiver’s permission for GSK to contact their child’s clinician for further information if required. However, should a parent/caregiver not choose to include theirs or their child’s clinician’s details on the AE form, they will be informed that the AE still has to be reported, but will be done so anonymously. Once they have been notified, Adelphi Values will report findings to the global drug safety pharmacovigilance department at GSK. Any completed forms will be sent to the designated contact at GSK within one business day of AV being made aware of the AE. Details of any AEs collected during the interviews will be described in the results section of the report.

Remember, an Adverse Event must be reported when the following are present:

**PREP** – Patient, Reporter, Event, Product

1. **A Patient or group of patients**

- Identifiers such as age, age group, birth date, gender, role, profession should be collected if available, if not the event should still be reported.

1. **An identifiable Reporting source**

- Information that identifies the reporter, establishing knowledge of the reportable event in an identifiable consumer. The reporter could be a nurse, pharmacist, patient or caregiver.

1. **An adverse Event**

- Description of at least one reportable event or product complaint, with as much detail as possible.

1. **GSK Product**

- At least one specific Sanofi product needs to be associated with the event.

An adverse event is:

***“An AE is any untoward medical occurrence in a patient or clinical investigation subject administered a pharmaceutical product and which does not necessarily have to have a causal relationship with this treatment. An AE can therefore be any unfavorable and unintended sign (including an abnormal laboratory finding, for example), symptom, or disease temporally associated with the use of a medicinal product, whether or not considered related to the medicinal product. (ICH-E2A)”***

**Interview introduction (5 minutes)**

**Objective**

To provide an overview of the study and interview structure, discuss confidentiality, anonymity and ask the participant’s permission to audio record the interview.

**Introductions**

- ***Thank the child for agreeing to participate in the study***
- Thank you for helping to us today!
- ***Introduce yourself as working on behalf of Adelphi Values***
- My name is (first name) and I work for a company called Adelphi Values. Adelphi Values is a company that works with companies that make medicines.
- ***Explain this particular project***
- Today we would like to find out what your asthma (OR term provided by parent/caregiver) is like. We are going to ask you some questions about what it is like to have asthma and we are going to look at some pictures that show what it is like to have asthma.
- If you find any questions hard to answer, or you don’t know what they mean, that is okay. ***There is no right or wrong answer;*** we just want to know about what life with asthma is like for you.
- ***Reassure the participant of confidentiality***
- Everything we talk about today will be between you, me, your mum/dad (caregiver) and people at Adelphi Values.
- ***Audio taping the interview***
- We will record [show recorder] our talk today so we can remember everything you say. Is that okay?
- Please try to speak in a loud voice so that we can hear what you say.
- You can stop at any point if you do not want to keep talking to us.

**Consent**

- ***At this point, turn on the tape recorder and say/ask the participant the following:***
- Do you agree to talk to me today?
- Do you agree to record this talk?
- Do you have any questions at this point?

***If the child responds “no”, clarify whether or not they wish to take part.***

- Just to check, do you want to take part in our chat today?

1. **If they do want to take part, explain that they must say they agree to participate on the recording, and that this won’t affect their right to withdraw later.**

- Ok, great. If you do want to take part, we need you to agree to us having our talk recorded today. However, you can stop our chat at any time.

1. **If they no longer want to take part or do not agree to have the interview audio recorded, thank them for their time but explain they cannot take part.**

**If the child asks any questions** **about the study**, and you know the answer, do your best to respond. For instance if the child asks questions about confidentiality, or how long the interview will take, you should answer these. However if you are not sure of the answer do not guess. If they ask a question relating to the study that you are not sure of, explain to them that you will find out the answer and that someone will get back to them as soon as possible.

If they ask any questions relating to asthma, explain that you are not a doctor, and that they should ask their doctor.

**Cognitive interviews (25 minutes)**

**Response scale 1 (A, B, C) (10 minutes)**

**Ranking exercise (2 minutes): *Provide the participant with the four image response options in a mixed-up order***

- Look at these four pictures. Please put them in order under each of the numbers 1 to 4. There are no right or wrong answers.

| 1  Bad asthma | 2 | 3 | 4  Good asthma |
| --- | --- | --- | --- |
|  |  |  |  |

1. **Can you please explain to me what each picture means?**
2. ***(FOCUS THE CHILD’S ATTENTION TO THE 1st AND 4th PICTURE).*** **Can you tell me the difference between picture 1 and picture 4?**

- *Can you tell me the difference between picture 2 and picture 3?*
- *Why did you put the pictures in that order?*

**Cognitive debriefing of the scale (8 minutes): Go to the device and introduce the questionnaire to the child**

- I am going to ask you to look at four questions about your asthma. I’m going to read each question to you and then you pick a picture for each one.

1. *(READ ALOUD ITEM 1 ON THE DEVICE -* *How is your asthma today?)* **What picture did you pick?**

- *Tell me about a bad day with your asthma? What picture would you pick on a bad day with your asthma?*
- *Tell me about a good day with your asthma? What picture would you pick on a good day with your asthma?*
- *(FOCUS THE CHILD’S ATTENTION TO THE GOOD DAY AND BAD DAY PICTURES). Can you tell me the difference between picture 1 and picture 4?*
- *If your asthma went from picture 1 to picture 2, what would be different about your asthma?*

1. *(READ ALOUD ITEM 2 ON THE DEVICE – How much of a problem is your asthma when…?)* **What picture did you pick?**

- *What games and sports do you like to play?*
- *Tell me about what games and sports you can play on a good day with your asthma? What picture would you pick on a good day with your asthma?*
- *Tell me about what games and sports you can play on a bad day with your asthma? What picture would you pick on a bad day with your asthma?*
- *(FOCUS THE CHILD’S ATTENTION TO THE GOOD DAY AND BAD DAY PICTURES). Can you tell me the difference between picture X and picture X?*
- *If your asthma went from picture 4 to picture 3, what would be different about your asthma?*

1. *(**READ ALOUD ITEM* *3 ON THE DEVICE – Do you cough because of your asthma?)* **What picture did you pick?**

- *How does your cough feel on a bad day? What picture would you pick?*
- *How does your cough feel on a good day? What picture would you pick?*
- *(FOCUS THE CHILD’S ATTENTION TO THE GOOD DAY AND BAD DAY PICTURES). Can you tell me the difference between picture X and picture X?*
- *If your asthma went from picture 2 to picture 3, what would be different about your asthma?*

1. *(READ ALOUD ITEM* *4 ON THE DEVICE – Do you wake up during the night….?)* **What picture did you pick?**

- *Tell me about a bad night with your asthma? What picture would you pick?*
- *Tell me about a good night with your asthma? What picture would you pick?*
- *(FOCUS THE CHILD’S ATTENTION TO THE GOOD DAY AND BAD DAY PICTURES). Can you tell me the difference between picture X and picture X?*
- *If your asthma went from picture 4 to picture 3, what would be different about your asthma?*

1. **What do you like about the pictures?**
2. **What don’t you like about the pictures?**
3. **If you could make the pictures better, what would you change?**

- *Why would these changes make the pictures better?*
- *Would these changes make it easier to answer the questions? Tell me about that.*

**Response scale 2 (A, B or C)**

**Ranking exercise (2 minutes): *Provide the participant with the four image response options in a mixed up order***

- Look at these four pictures. Please put them in order under each of the numbers 1 to 4. There are no right or wrong answers.

| 1  Bad asthma | 2 | 3 | 4  Good asthma |
| --- | --- | --- | --- |
|  |  |  |  |

1. **Can you please explain to me what each picture means?**
2. ***(FOCUS THE CHILD’S ATTENTION TO THE 1st AND 4th PICTURE).*** **Can you tell me the difference between picture 1 and picture 4?**

- *Can you tell me the difference between picture 2 and picture 3?*
- *Why did you put the pictures in that order?*

**Cognitive debriefing of the scale (8 mins): Introduce the second version of the questionnaire**

- I am going to show you the same four questions about your asthma. But this time the questions have different pictures for you to pick from. We want to find out what you think of the new pictures.

1. *(READ ALOUD ITEM 1 ON THE DEVICE -* *How is your asthma today?)* **What picture did you pick?**

- *Tell me about a bad day with your asthma? What picture would you pick on a bad day with your asthma?*
- *Tell me about a good day with your asthma? What picture would you pick on a good day with your asthma?*
- *(FOCUS THE CHILD’S ATTENTION TO THE GOOD DAY AND BAD DAY PICTURES). Can you tell me the difference between picture 1 and picture 4?*
- *If your asthma went from picture 1 to picture 2, what would be different about your asthma?*

1. *(READ ALOUD ITEM 2 ON THE DEVICE – How much of a problem is your asthma when…?)* **What picture did you pick?**

- *What games and sports do you like to play?*
- *Tell me about what games and sports you can play on a good day with your asthma? What picture would you pick on a good day with your asthma?*
- *Tell me about what games and sports you can play on a bad day with your asthma? What picture would you pick on a bad day with your asthma?*
- *(FOCUS THE CHILD’S ATTENTION TO THE GOOD DAY AND BAD DAY PICTURES). Can you tell me the difference between picture X and picture X?*
- *If your asthma went from picture 4 to picture 3, what would be different about your asthma?*

1. *(READ ALOUD ITEM 3 ON THE DEVICE – Do you cough because of your asthma?)* **What picture did you pick?**

- *How does your cough feel on a bad day? What picture would you pick?*
- *How does your cough feel on a good day? What picture would you pick?*
- *(FOCUS THE CHILD’S ATTENTION TO THE GOOD DAY AND BAD DAY PICTURES). Can you tell me the difference between picture X and picture X?*
- *If your asthma went from picture 2 to picture 3, what would be different about your asthma?*

1. *(READ ALOUD ITEM* *4 ON THE DEVICE – Do you wake up during the night….?)* **What picture did you pick?**

- *Tell me about a bad night with your asthma? What picture would you pick?*
- *Tell me about a good night with your asthma? What picture would you pick?*
- *(FOCUS THE CHILD’S ATTENTION TO THE GOOD DAY AND BAD DAY PICTURES). Can you tell me the difference between picture X and picture X?*
- *If your asthma went from picture 4 to picture 3, what would be different about your asthma?*

1. **What do you like about the pictures?**
2. **What don’t you like about the pictures?**
3. **If you could make the pictures better, what would you change?**

- *Why would these changes make the pictures better?*
- *Would these changes make it easier to answer the questions, why?*

**Cognitive questions- comparison of scales (3 minutes)**

- We have looked at face pictures and shape pictures today to answer questions about your asthma.

1. **What pictures did you like the most?**

- *What pictures were easy to pick an answer? Tell me about that.*
- *What pictures were difficult to pick answer. Tell me about that.*

**Cognitive questions- original C-ACT (2 minutes)**

***Lay out the original C-ACT and introduce the draft questionnaire to the participant***

- I am going to ask you to look at these pictures for each question.

1. **What do you like about these pictures? What do you not like about these pictures?**

- *Are these pictures better to describe your asthma?*
- *Are these pictures worse to describe your asthma?*
- *Would it be easy or difficult to pick an answer with these pictures? Tell me about that.*
- We have now finished all the questions. Thank you for answering so well. Do you have any questions before we finish?

**Appendix 1: Copy of the current C-ACT**


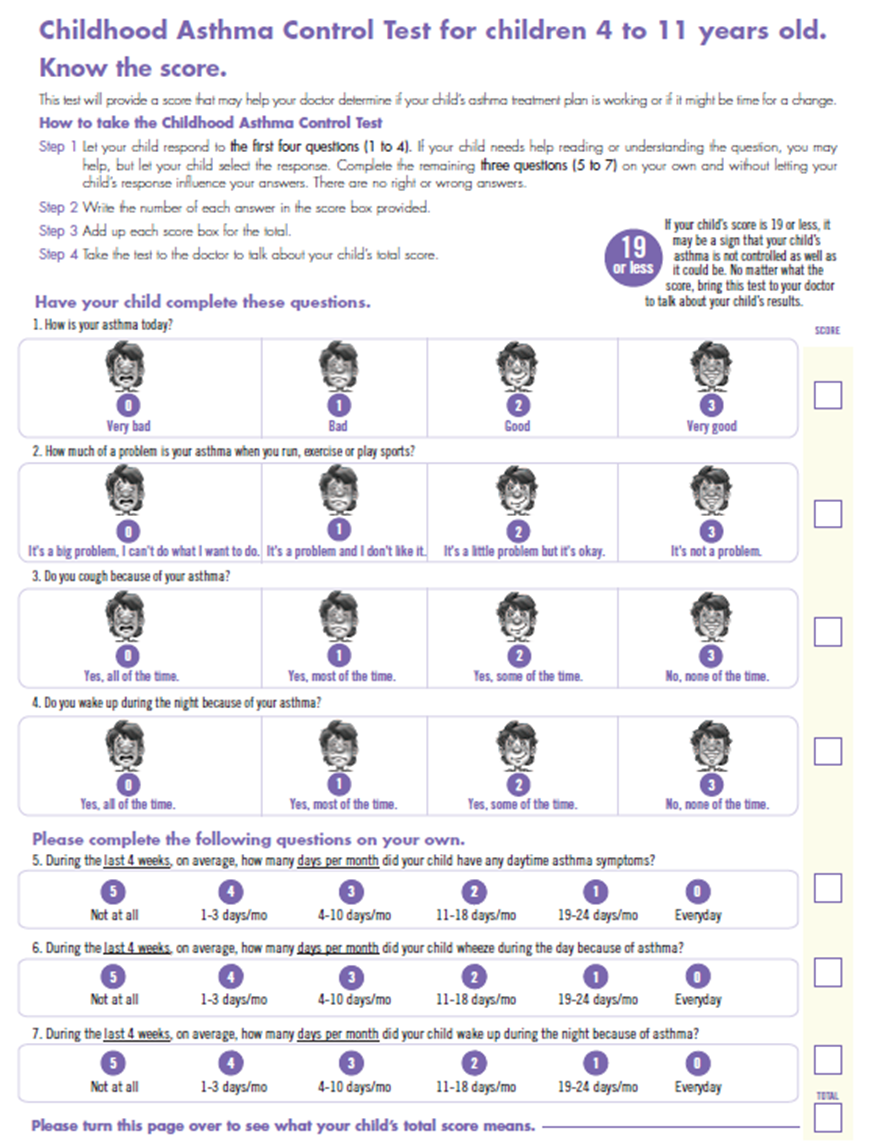


**Appendix 2: Adverse Event form**


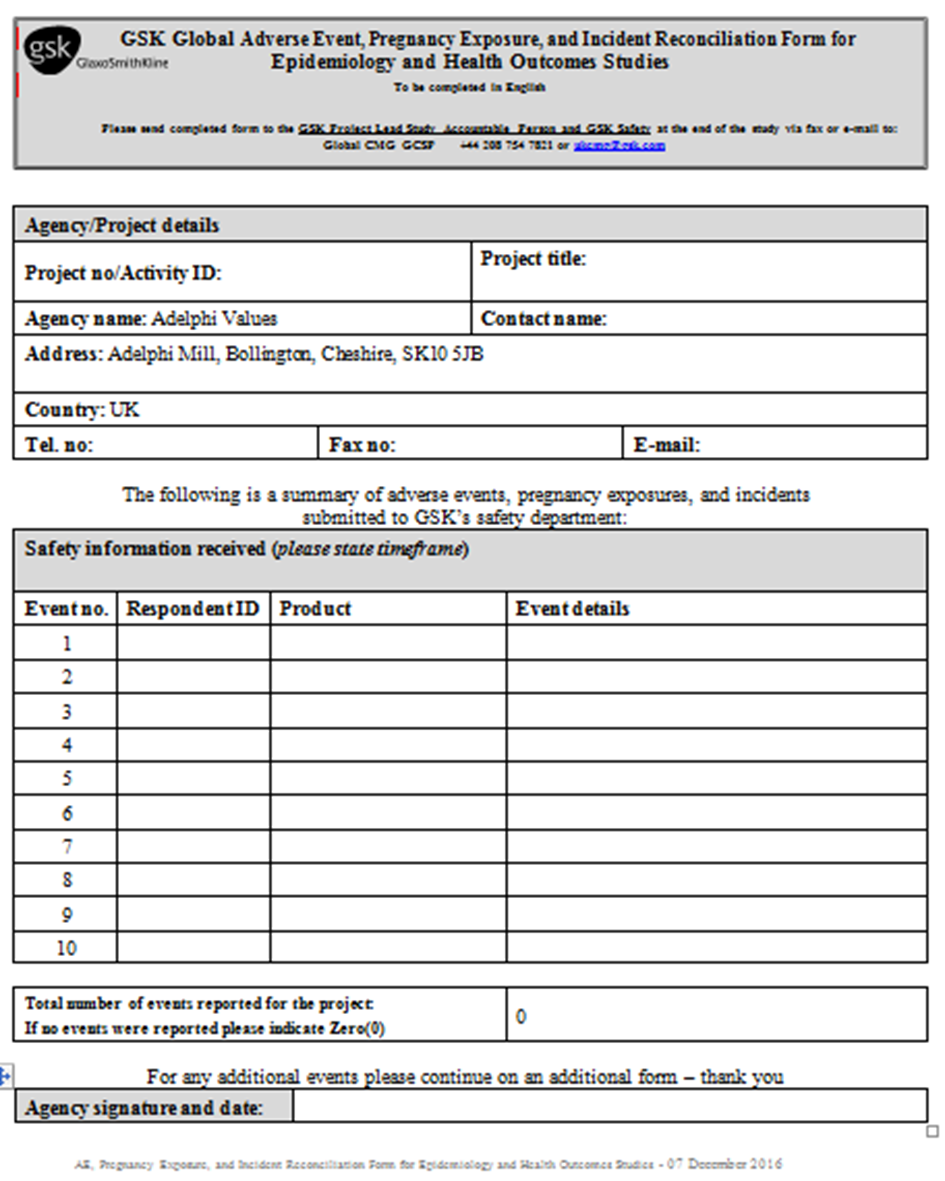


| G | K | 8 | 0 | 3 | 3 | A |  |  |  |
| --- | --- | --- | --- | --- | --- | --- | --- | --- | --- |
| Project Code | | | | | | | Subject ID | | |

**Supplementary File 2** Round 2 Interview Guide

| Date of interview: |  | / |  | / |  |
| --- | --- | --- | --- | --- | --- |
|  | Day |  | Month |  | Year |

| Location of interview: |  | , |  |
| --- | --- | --- | --- |
|  | City |  | Country |

| Name of interviewer: |  |  |  |
| --- | --- | --- | --- |
|  |  |  |  |

**Interview objectives**

The overall objective of the project is to develop and confirm the content validity and qualitative equivalency of a modified version of the C-ACT (see Appendix 1), specifically a version with updated image response options for the child-completed items (1-4). It is intended that the interviews will be conducted in two rounds. Participants will complete the modified versions of the C-ACT on an electronic hand-held device.

The specific objectives of the first round of cognitive interviews are:

- To evaluate the acceptability, relevance and understanding of up to three modified image response scales
- To assess the usability of the modified image response scales on an electronic mode of administration

The specific objective of the second round of cognitive interviews are:

- To confirm content validity and qualitative equivalency of the modified image response scale(s) with the original version
- To assess the usability of the modified image response scales on an electronic mode of administration

**Overview of the interview**

The interview should be conducted as follows:

1. **Introduction to the study**: Introduce Adelphi Values to the participant. Explain the objectives and the process of the interview.
2. **Consent:** Ensure written informed consent to participate has been obtained prior to the beginning of the interview. Also obtain the participant’s verbal consent for the interview to be audio-recorded.
3. **Interview:**

The interview is divided into the following sections:

- Introduction to the study and consent (5 minutes)
- Image response scale A (9 minutes)
  - Ranking exercise
  - Cognitive debriefing of the scale
- Original C-ACT (4 minutes)
  - Qualitative equivalence of new response scale (A or B) and original C-ACT
- Image response scale B (9 minutes)
  - Ranking exercise
  - Cognitive debriefing of the scale
- Comparison of image response scale A and scale B (3 minutes)

In total, the interview should take approximately **30 minutes** (not including travel and scheduling time).

**Role of the interviewer and instructions**

**The role of the interviewer before the interview**

1. Check the screener to confirm that the child is eligible to take part.
2. Check the caregiver/child (only those aged >10 years old) has provided written informed consent/assent. All children must provide verbal consent for the interview to be audio recorded.
3. Check the ePRO is charged.
4. Print a copy of:
   1. Response scales A and B (see Appendix 1)
   2. The original C-ACT (see Appendix 2)
   3. Additional questions for the parent/caregiver to complete (see Appendix 3)

**Checks** **with caregivers prior to the child interview**

**Before the interview starts, briefly take the child’s parent/caregiver to the side and ask the following to aid you in probing the child**

1. **How does [the child] refer to their asthma?**

Does s/he just talk about “asthma” or “breathing problems”?

1. **How does [the child] talk about their symptoms?**

What words does s/he use to talk about breathing difficulty, breathlessness or shortness of breath?

What words do you use to talk about coughing?

**The role of the interviewer during the interview**

- **Digital Audio recording**: Ensure that the comments of the child throughout the interview are clearly recorded on the audio-recording. Audio recordings will be transcribed verbatim. Check the volume settings and positioning of the audio recorder to ensure the clarity of the audio recording. Avoid rustling papers near the recorder or jostling the recorder during the interview as this will lead to inaudible responses.
- **Interview guide:** Make a written note in the interview guide of any non-verbal communication by the patient (e.g., vocalisations or facial expressions indicating rejection or acceptance of one of your comments; apparent affect such as confusion, frustration, annoyance, etc.). Comment on the non-verbal cue and invite the patient to explain his/her feelings to ensure you have properly interpreted the gesture.
- **Manging parent/caregiver:** If it is necessary for a parent/caregiver to be present in the interview, they will be asked to sit out of the child’s eye line and asked not to directly respond on behalf the child. However, the parent/caregiver can provide encouragement to the child or explain a question to them if necessary.
- **Record answers provided for response scale A and B**: During the interview record the answers provided by the participant for response scale A and B **on paper** using Appendix 1.
- **Completion of C-ACT:** During each interview ensure the child completes items 1-4 of the original C-ACT (**on paper**) using Appendix 2.

The role of the interviewer is to **listen** and **support:**

- Please do not give your own point of view
- Be patient and accept silence during open-ended questioning to make sure the participant has time to think about his/her response
- Help the participant stick to the topics intended in the guide
- Help the participant cross-reference and make connections that they would not do spontaneously
- Ask the participant to clarify any comments or gestures that you are not certain you understood

**The role of the interviewer after the interview**

- After each interview ensure the parent/caregiver completes items 5-7 of the original C-ACT (**on paper**) using Appendix 2.
- After each interview ensure the parent/caregiver completes the additional questions in Appendix 3 (**on paper**).

**Using the Interview Guide**

Throughout this guide we have coded the questions and instructions so that it is clear which questions are required and which are probes, to be used when necessary. The codes are as follows:

| UPPER CASE TEXT | **These are guidance notes for you (the interviewer)** |
| --- | --- |
| - ***Bold, italic, bulleted text*** | These are instructions to you (the interviewer) |
|  | These are statements that you read aloud word-for-word to the participant. |
| 1. **Bold text** | Questions prefixed by a specific number must be asked to all participants |
| - *Italic text* | All probes or follow up questions are bulleted – these do not necessarily need to be used, but are included to assist you in drawing further information from participants if needed. The probes vary in difficulty and follow a traffic light system, so that you (the interviewer) are able to easily identify probes which participants may find more difficult to understand. |
| - *Green italics* | If a participant understands the first question, ask this low ability follow up probe. |
| - *Amber italics* | If a participant understands the green italic probe, ask this middle ability follow up probe. |
| - *Red italics* | If a participant understands the amber italic probe, ask this high ability follow up probe. |

**Adverse Events**

**What should you do if an adverse event is mentioned?**

If during an interview, a child or patient/caregiver mentions an adverse event (AE) or product complaint related to a product of the sponsor, whether it is considered serious or not, this must be reported to the appropriate drug safety/pharmacovigilance department at the sponsor.

If the child or patient/caregiver mentions an adverse event that they or anyone else experienced while taking any GSK product the interviewer will complete an adverse event (AE) reporting form (See Appendix 4) and send to Adelphi Values via email (kate.sully@adelphivalues.com). The interviewer will complete the AE reporting form at the end of the interview that will capture information regarding the reportable event, the GSK product involved and the role of the person reporting the event. The AV trained interviewer will also seek the parent/caregiver’s permission for GSK to contact their child’s clinician for further information if required. However, should a parent/caregiver not choose to include theirs or their child’s clinician’s details on the AE form, they will be informed that the AE still has to be reported, but will be done so anonymously. Once they have been notified, Adelphi Values will report findings to the global drug safety pharmacovigilance department at GSK. Any completed forms will be sent to the designated contact at GSK within one business day of AV being made aware of the AE. Details of any AEs collected during the interviews will be described in the results section of the report.

Remember, an Adverse Event must be reported when the following are present:

**PREP** – Patient, Reporter, Event, Product

1. **A Patient or group of patients**

- Identifiers such as age, age group, birth date, gender, role, profession should be collected if available, if not the event should still be reported.

1. **An identifiable Reporting source**

- Information that identifies the reporter, establishing knowledge of the reportable event in an identifiable consumer. The reporter could be a nurse, pharmacist, patient or caregiver.

1. **An adverse Event**

- Description of at least one reportable event or product complaint, with as much detail as possible.

1. **GSK Product**

- At least one specific Sanofi product needs to be associated with the event.

An adverse event is:

***“An AE is any untoward medical occurrence in a patient or clinical investigation subject administered a pharmaceutical product and which does not necessarily have to have a causal relationship with this treatment. An AE can therefore be any unfavorable and unintended sign (including an abnormal laboratory finding, for example), symptom, or disease temporally associated with the use of a medicinal product, whether or not considered related to the medicinal product. (ICH-E2A)”***

**Interview introduction (5 minutes)**

**Objective**

To provide an overview of the study and interview structure, discuss confidentiality, anonymity and ask the participant’s permission to audio record the interview.

**Introductions**

- ***Thank the child for agreeing to participate in the study***
- Thank you for helping to us today!
- ***Introduce yourself as working on behalf of Adelphi Values***
- My name is (first name) and I work for a company called Adelphi Values. Adelphi Values is a company that works with companies that make medicines.
- ***Explain this particular project***
- Today we would like to find out what your asthma (OR term provided by parent/caregiver) is like. We are going to ask you some questions about what it is like to have asthma and we are going to look at some pictures that show what it is like to have asthma.
- If you find any questions hard to answer, or you don’t know what they mean, that is okay. ***There is no right or wrong answer;*** we just want to know about what life with asthma is like for you.
- ***Reassure the participant of confidentiality***
- Everything we talk about today will be between you, me, your mum/dad (caregiver) and people at Adelphi Values.
- ***Audio taping the interview***
- We will record [show recorder] our talk today so we can remember everything you say. Is that okay?
- Please try to speak in a loud voice so that we can hear what you say.
- You can stop at any point if you do not want to keep talking to us.

**Consent**

- ***At this point, turn on the tape recorder and say/ask the participant the following:***
- Do you agree to talk to me today?
- Do you agree to record this talk?
- Do you have any questions at this point?

***If the child responds “no”, clarify whether or not they wish to take part.***

- Just to check, do you want to take part in our chat today?

1. **If they do want to take part, explain that they must say they agree to participate on the recording, and that this won’t affect their right to withdraw later.**

- Ok, great. If you do want to take part, we need you to agree to us having our talk recorded today. However, you can stop our chat at any time.

1. **If they no longer want to take part or do not agree to have the interview audio recorded, thank them for their time but explain they cannot take part.**

**If the child asks any questions** **about the study**, and you know the answer, do your best to respond. For instance if the child asks questions about confidentiality, or how long the interview will take, you should answer these. However if you are not sure of the answer do not guess. If they ask a question relating to the study that you are not sure of, explain to them that you will find out the answer and that someone will get back to them as soon as possible.

If they ask any questions relating to asthma, explain that you are not a doctor, and that they should ask their doctor.

**Cognitive interviews (25 minutes)**

**Response scale 1 (A or B) (9 minutes)**

**Ranking exercise (2 minutes): *Provide the participant with the four image response options in a mixed-up order***

- Look at these four pictures. Please put them in order under each of the numbers 1 to 4 – 1 is bad asthma and 4 is good asthma. There are no right or wrong answers.

| 1  Bad asthma | 2 | 3 | 4  Good asthma |
| --- | --- | --- | --- |
|  |  |  |  |

1. **Can you please explain to me what each picture means?**
2. ***(FOCUS THE CHILD’S ATTENTION TO THE 1st AND 4th PICTURE).*** **Can you tell me the difference between picture 1 and picture 4?**

- *Can you tell me the difference between picture 2 and picture 3?*
- *Why did you put the pictures in that order?*

**Cognitive debriefing of the scale (7 minutes): Go to the device and introduce the questionnaire to the child**

- I am going to ask you to look at four questions about your asthma. I’m going to read each question to you and then you pick a picture for each one.

1. *(READ ALOUD ITEM 1 ON THE DEVICE -* *How is your asthma today?)* **What picture did you pick?**

- *Tell me about a bad day with your asthma? What picture would you pick on a bad day with your asthma?*
- *Tell me about a good day with your asthma? What picture would you pick on a good day with your asthma?*
- *(FOCUS THE CHILD’S ATTENTION TO THE GOOD DAY AND BAD DAY PICTURES). Can you tell me the difference between picture 1 and picture 4?*
- *If your asthma went from picture 1 to picture 2, what would be different about your asthma?*

1. *(READ ALOUD ITEM 2 ON THE DEVICE – How much of a problem is your asthma when…?)* **What picture did you pick?**

- *What games and sports do you like to play?*
- *Tell me about what games and sports you can play on a good day with your asthma? What picture would you pick on a good day with your asthma?*
- *Tell me about what games and sports you can play on a bad day with your asthma? What picture would you pick on a bad day with your asthma?*
- *(FOCUS THE CHILD’S ATTENTION TO THE GOOD DAY AND BAD DAY PICTURES). Can you tell me the difference between picture X and picture X?*
- *If your asthma went from picture 4 to picture 3, what would be different about your asthma?*

1. *(READ ALOUD ITEM 3 ON THE DEVICE – Do you cough because of your asthma?)* **What picture did you pick?**

- *How does your cough feel on a bad day? What picture would you pick?*
- *How does your cough feel on a good day? What picture would you pick?*
- *(FOCUS THE CHILD’S ATTENTION TO THE GOOD DAY AND BAD DAY PICTURES). Can you tell me the difference between picture X and picture X?*
- *If your asthma went from picture 2 to picture 3, what would be different about your asthma?*

1. *(READ ALOUD ITEM* *4 ON THE DEVICE – Do you wake up during the night….?)* **What picture did you pick?**

- *Tell me about a bad night with your asthma? What picture would you pick?*
- *Tell me about a good night with your asthma? What picture would you pick?*
- *(FOCUS THE CHILD’S ATTENTION TO THE GOOD DAY AND BAD DAY PICTURES). Can you tell me the difference between picture X and picture X?*
- *If your asthma went from picture 4 to picture 3, what would be different about your asthma?*

1. **What do you like about the pictures?**
2. **What don’t you like about the pictures?**
3. **If you could make the pictures better, what would you change?**

- *Why would these changes make the pictures better?*

**Cognitive questions- original C-ACT (4 minutes)**

***Put the paper version of scale A or B (simple face or circle) with the completed responses*** ***in front of the participant.***

***Put the paper version of the original C-ACT in front of the child and introduce the original C-ACT to the participant.***

- We have just looked at X (simple face or circle) pictures to answer questions about your asthma today (point to paper version in front of the child).
- I am going to ask you to look at these different pictures for the same questions about your asthma (point to paper version of original C-ACT in front of the child).

1. ***What picture would you pick for question 1, ‘How is your asthma today’?***
2. ***What picture would you pick for question 2, ‘How much of a problem is your asthma when you run, exercise or play sports?’***
3. ***What picture would you pick for question 3, ‘Do you cough because of your asthma?’***
4. ***What picture would you pick for question 4, ‘Do you wake up during the night because of your asthma?’***

***If the child picks the same responses for scale A or B (simple face or circle) and the original C-ACT, ask the following:***

- *Why did you pick the same answers using these pictures?*

***If the child picks different responses for scale A or B (simple face or circle) and the original C-ACT, ask the following:***

- *For this question, on this one you chose this picture, but on this one you chose this one. Why did you pick different answers using these pictures? Do you think these pictures mean better/worse asthma?*

1. **What pictures best describe your asthma? (point to the original C-ACT images and the simple faces or circles)**

- *If yes, what makes these pictures better?*
- *If no, what makes these pictures the same?*

**Response scale 2 (A or B) (9 minutes)**

**Ranking exercise (2 minutes): *Provide the participant with the four image response options in a mixed up order***

- Look at these four pictures. Please put them in order under each of the numbers 1 to 4. There are no right or wrong answers.

| 1  Bad asthma | 2 | 3 | 4  Good asthma |
| --- | --- | --- | --- |
|  |  |  |  |

1. **Can you please explain to me what each picture means?**
2. ***(FOCUS THE CHILD’S ATTENTION TO THE 1st AND 4th PICTURE).*** **Can you tell me the difference between picture 1 and picture 4?**

- *Can you tell me the difference between picture 2 and picture 3?*
- *Why did you put the pictures in that order?*

**Cognitive debriefing of the scale (7 mins): Introduce the second version of the questionnaire**

- I am going to show you the same four questions about your asthma. But this time the questions have different pictures for you to pick from. We want to find out what you think of the new pictures.

1. *(READ ALOUD ITEM 1 ON THE DEVICE -* *How is your asthma today?)* **What picture did you pick?**

- *Tell me about a bad day with your asthma? What picture would you pick on a bad day with your asthma?*
- *Tell me about a good day with your asthma? What picture would you pick on a good day with your asthma?*
- *(FOCUS THE CHILD’S ATTENTION TO THE GOOD DAY AND BAD DAY PICTURES). Can you tell me the difference between picture 1 and picture 4?*
- *If your asthma went from picture 1 to picture 2, what would be different about your asthma?*

1. *(READ ALOUD ITEM 2 ON THE DEVICE – How much of a problem is your asthma when…?)* **What picture did you pick?**

- *What games and sports do you like to play?*
- *Tell me about what games and sports you can play on a good day with your asthma? What picture would you pick on a good day with your asthma?*
- *Tell me about what games and sports you can play on a bad day with your asthma? What picture would you pick on a bad day with your asthma?*
- *(FOCUS THE CHILD’S ATTENTION TO THE GOOD DAY AND BAD DAY PICTURES). Can you tell me the difference between picture X and picture X?*
- *If your asthma went from picture 4 to picture 3, what would be different about your asthma?*

1. *(READ ALOUD ITEM 3 ON THE DEVICE – Do you cough because of your asthma?)* **What picture did you pick?**

- *How does your cough feel on a bad day? What picture would you pick?*
- *How does your cough feel on a good day? What picture would you pick?*
- *(FOCUS THE CHILD’S ATTENTION TO THE GOOD DAY AND BAD DAY PICTURES). Can you tell me the difference between picture X and picture X?*
- *If your asthma went from picture 2 to picture 3, what would be different about your asthma?*

1. *(READ ALOUD ITEM* *4 ON THE DEVICE – Do you wake up during the night….?)* **What picture did you pick?**

- *Tell me about a bad night with your asthma? What picture would you pick?*
- *Tell me about a good night with your asthma? What picture would you pick?*
- *(FOCUS THE CHILD’S ATTENTION TO THE GOOD DAY AND BAD DAY PICTURES). Can you tell me the difference between picture X and picture X?*
- *If your asthma went from picture 4 to picture 3, what would be different about your asthma?*

1. **What do you like about the pictures?**
2. **What don’t you like about the pictures?**
3. **If you could make the pictures better, what would you change?**

- *Why would these changes make the pictures better?*

**Cognitive questions- comparison of scales (3 minutes)**

***Put the paper version of scale A and B with the completed responses*** ***in front of the participant.***

- We have looked at face pictures (point to completed paper version of face questionnaire) and circle pictures (point to completed paper version of circle questionnaire) today to answer questions about your asthma.

1. **What pictures did you like the most?**

- *What pictures helped you answer the questions best?*
- *What pictures were easy to pick an answer? Tell me about that.*
- *What pictures were difficult to pick answer. Tell me about that.*
- *What pictures were best for describing your asthma?*
- We have now finished all the questions. Thank you for answering so well. Do you have any questions before we finish?

**Appendix 1: Copy of response scale A and B**

**Response scale A**


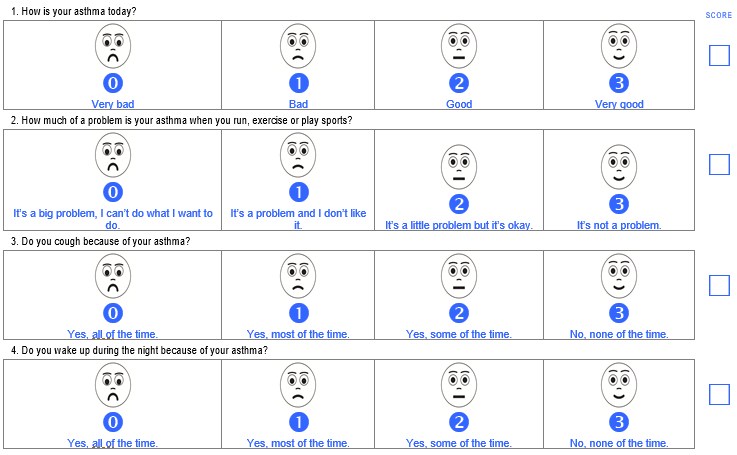


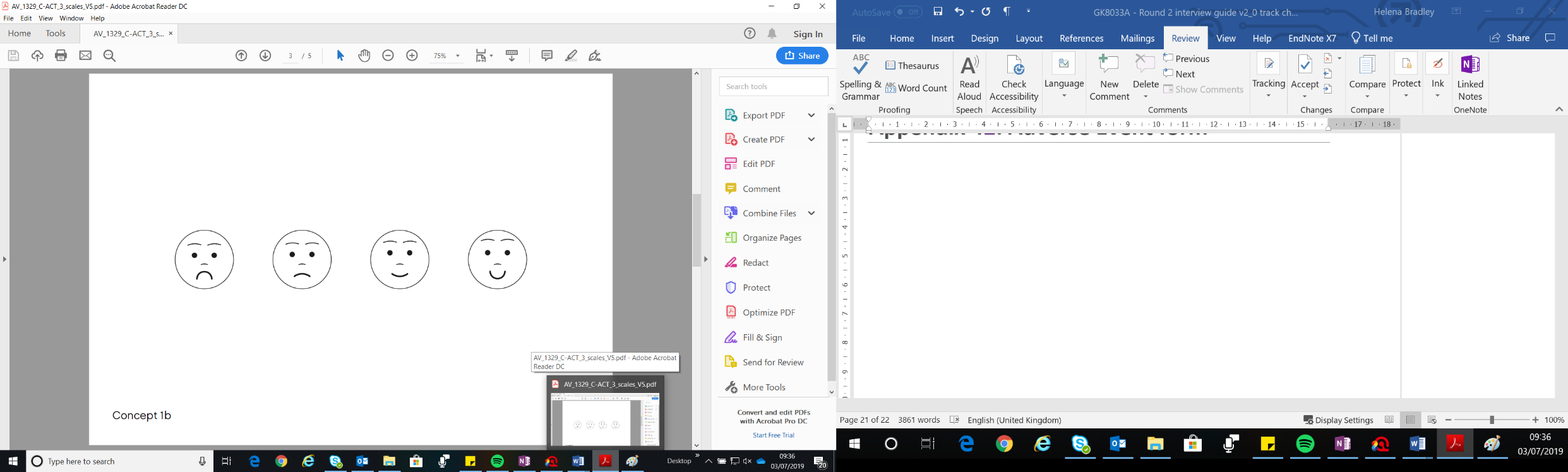

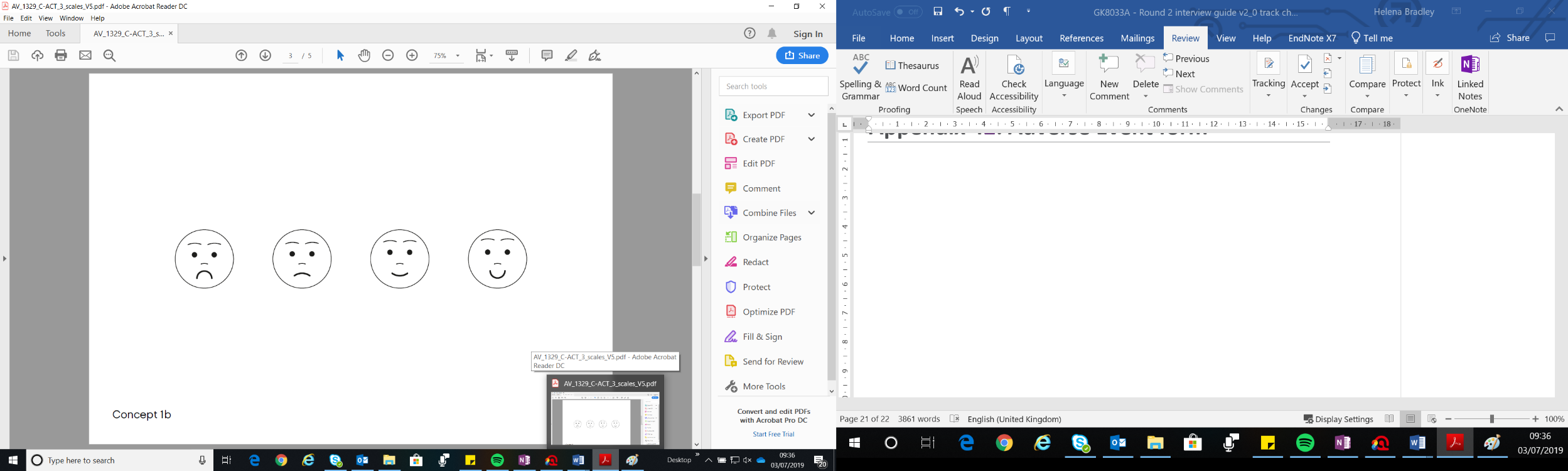

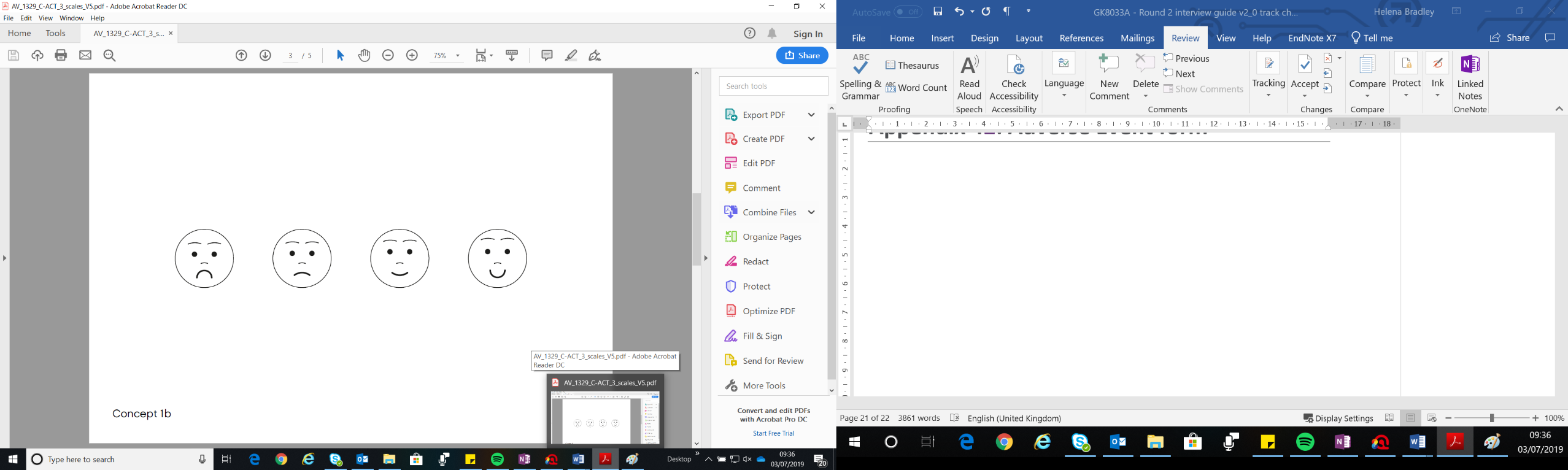

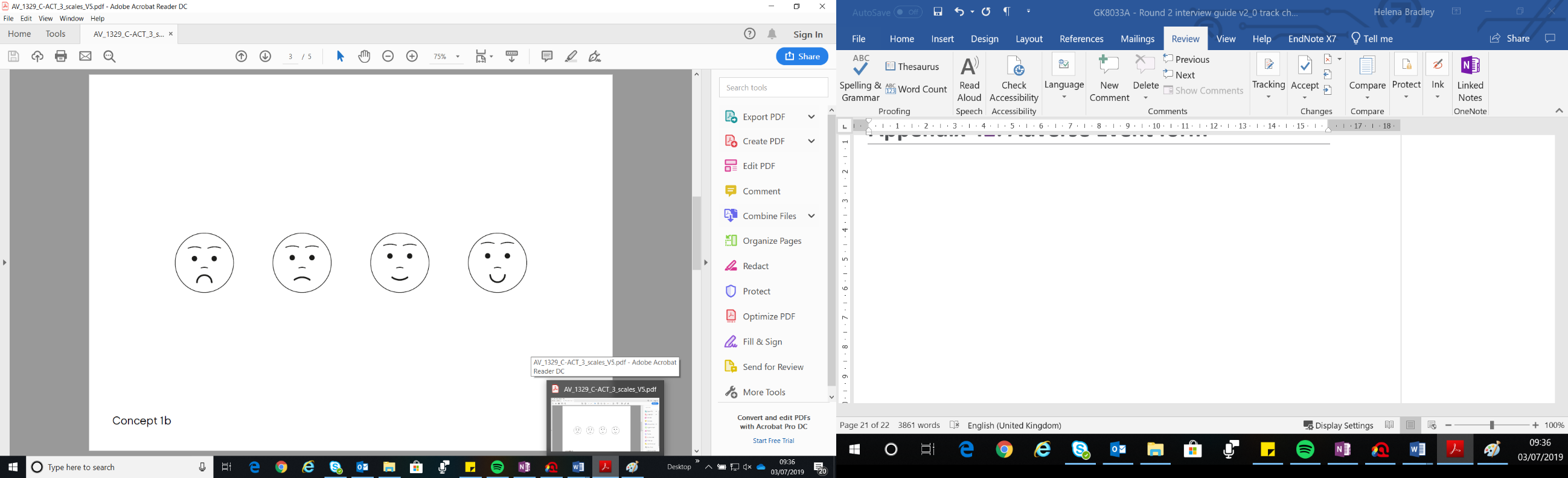

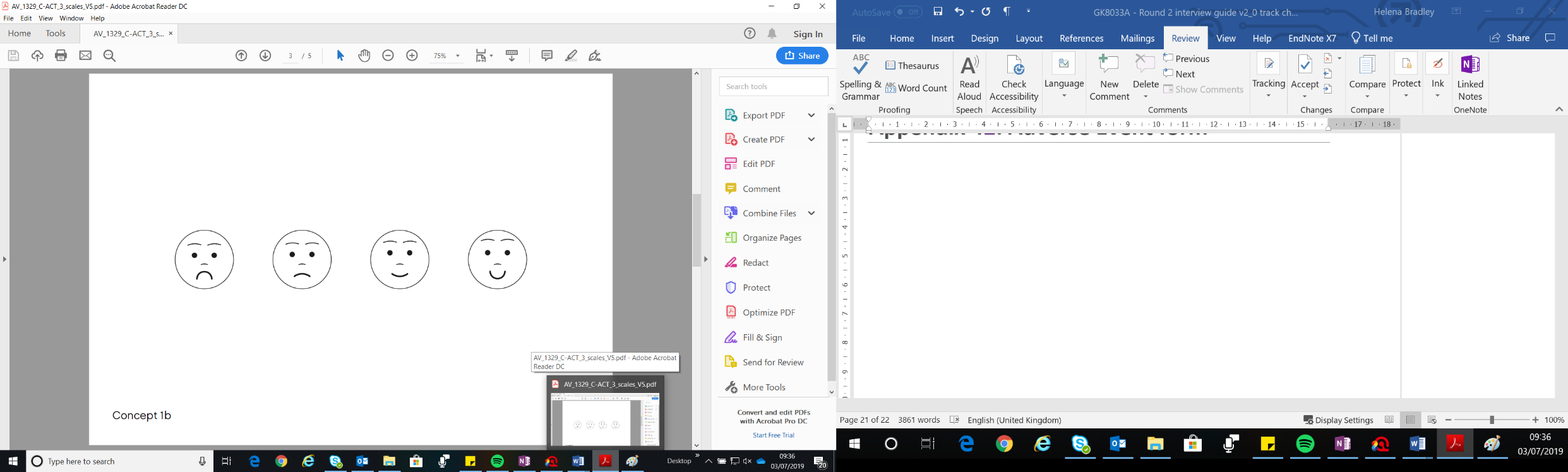

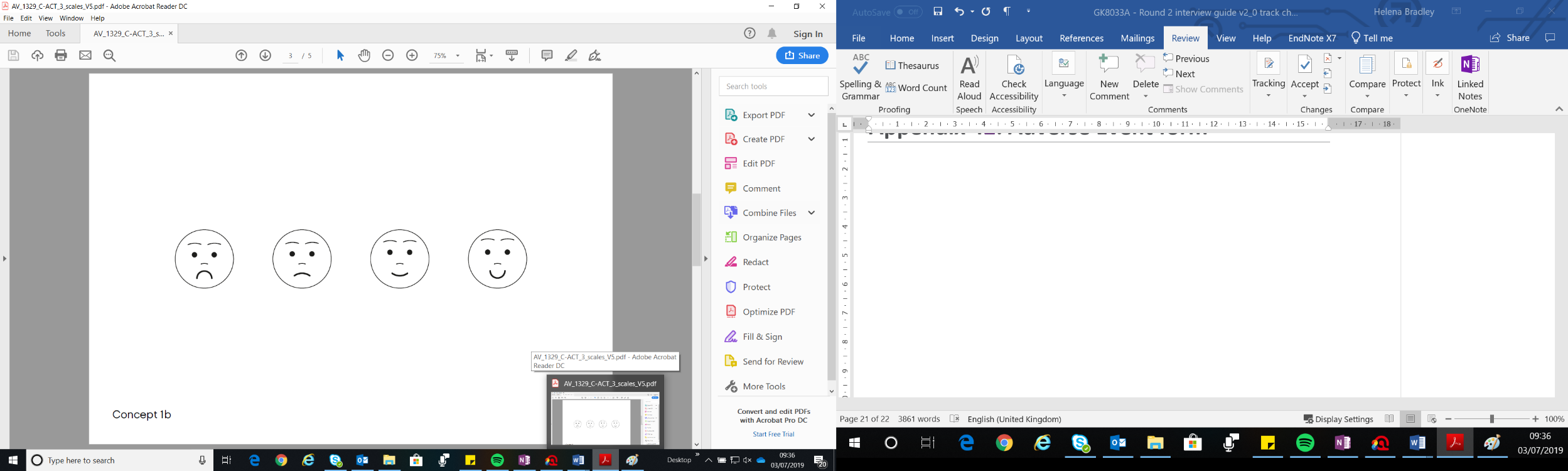

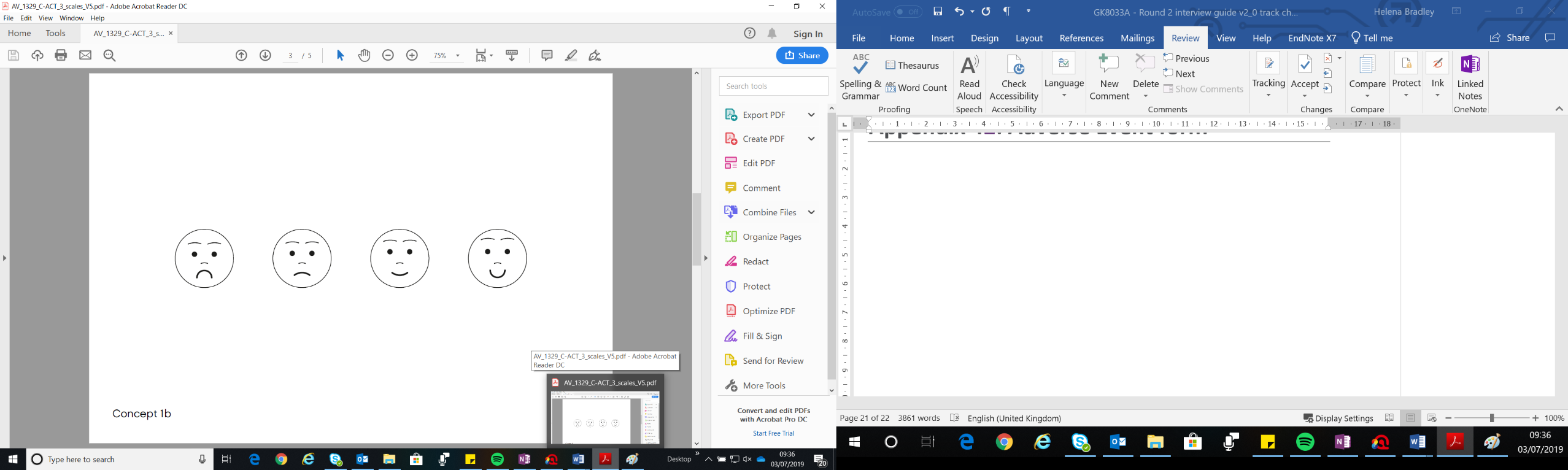

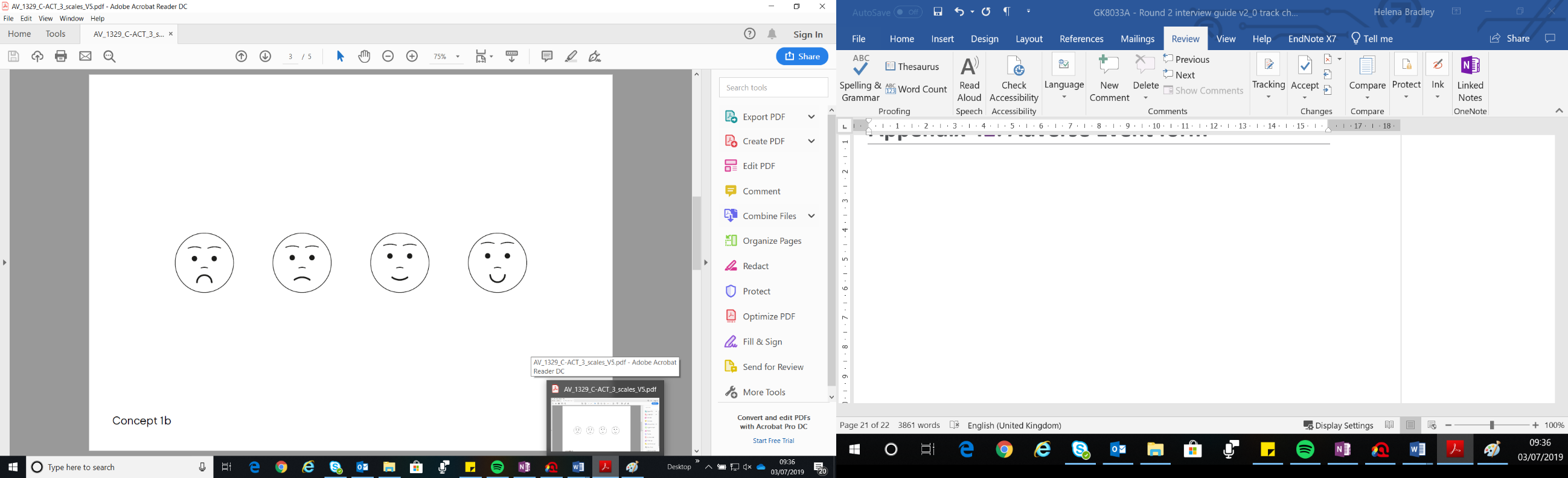

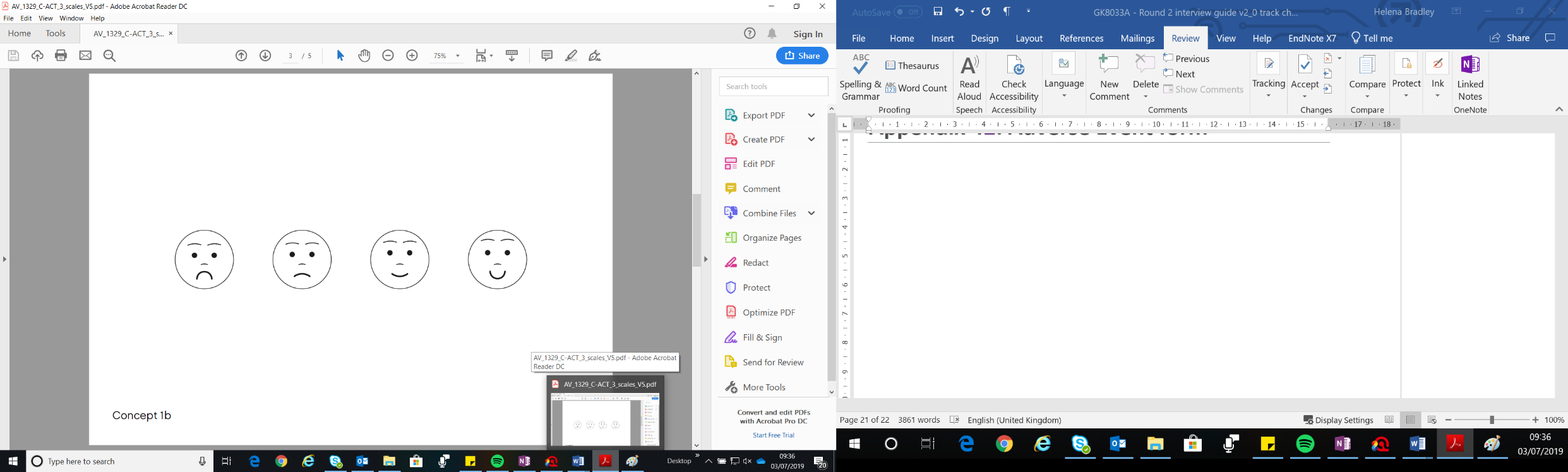

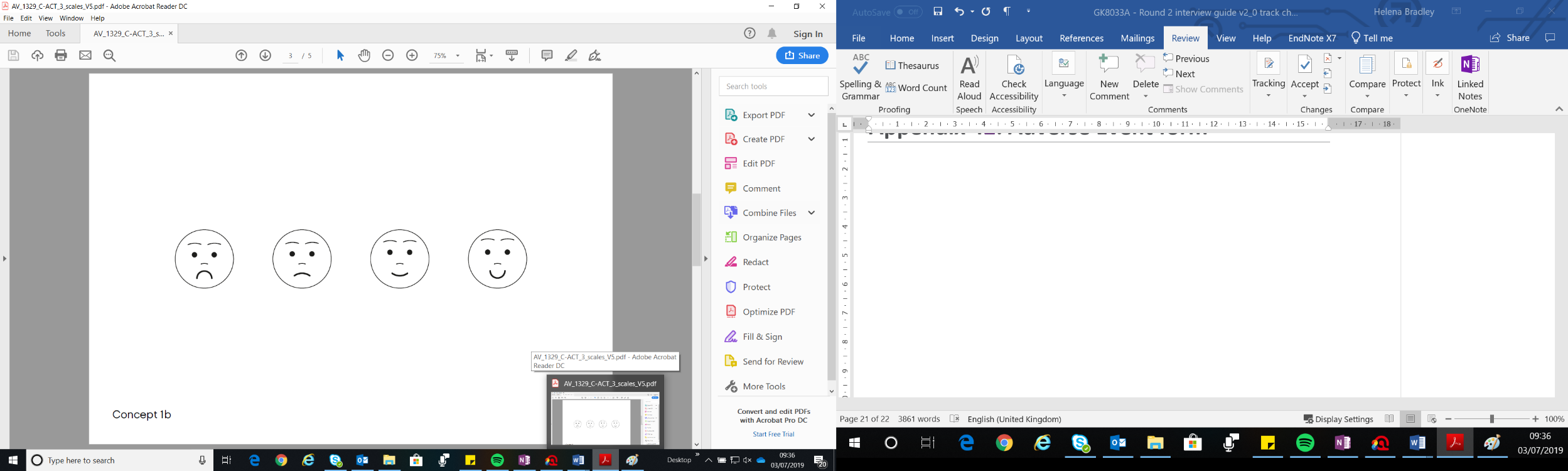

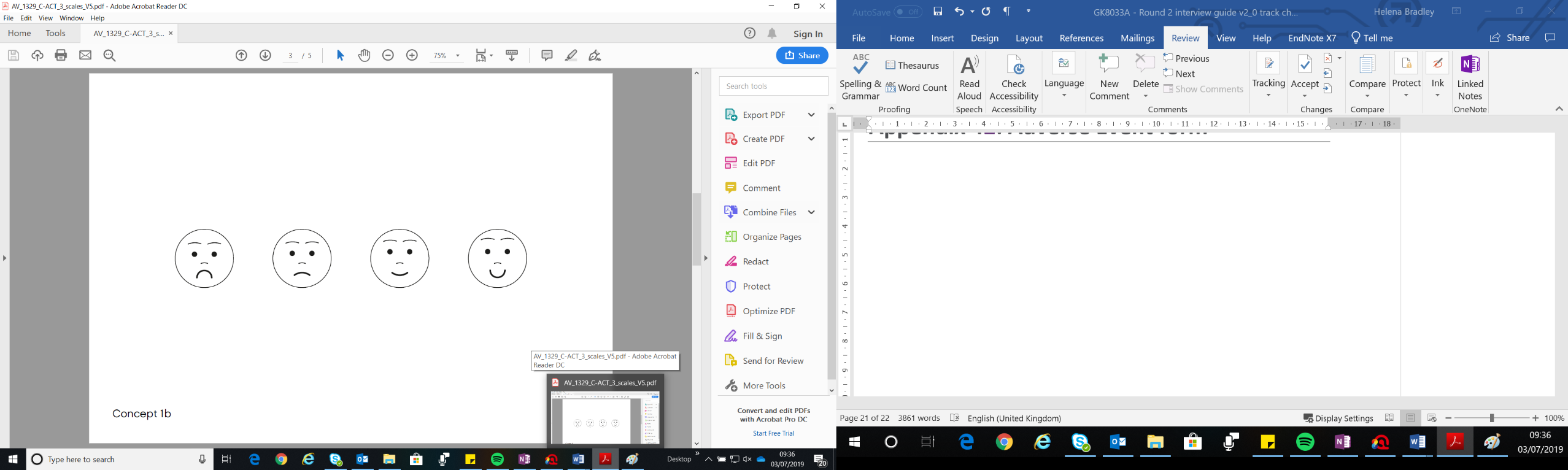

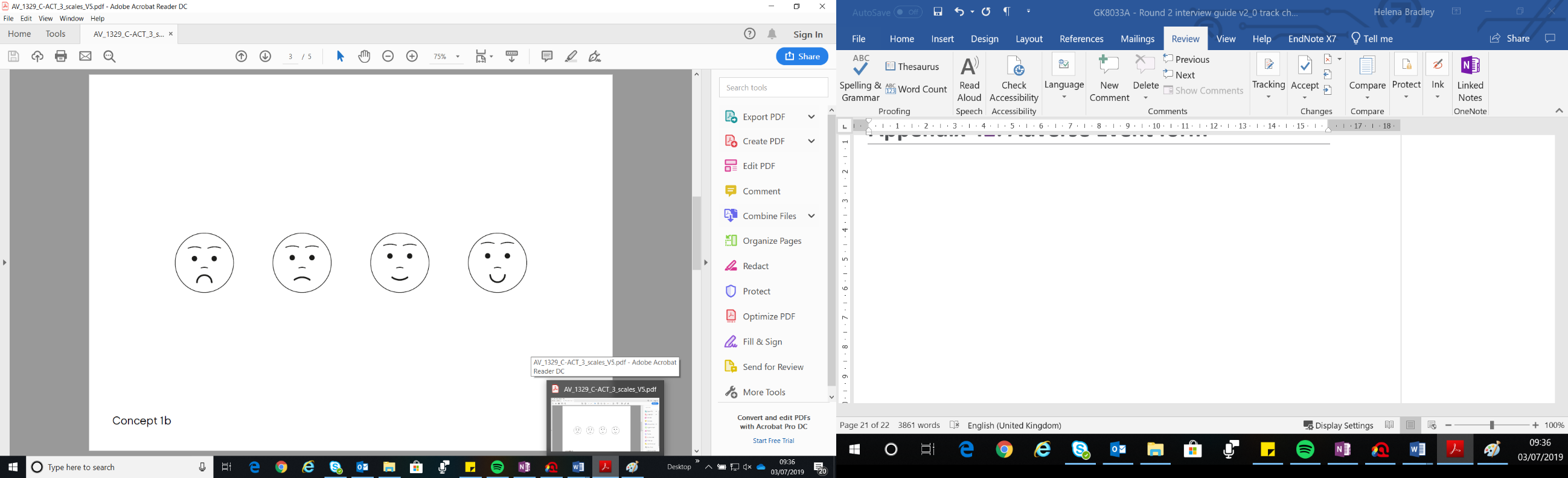

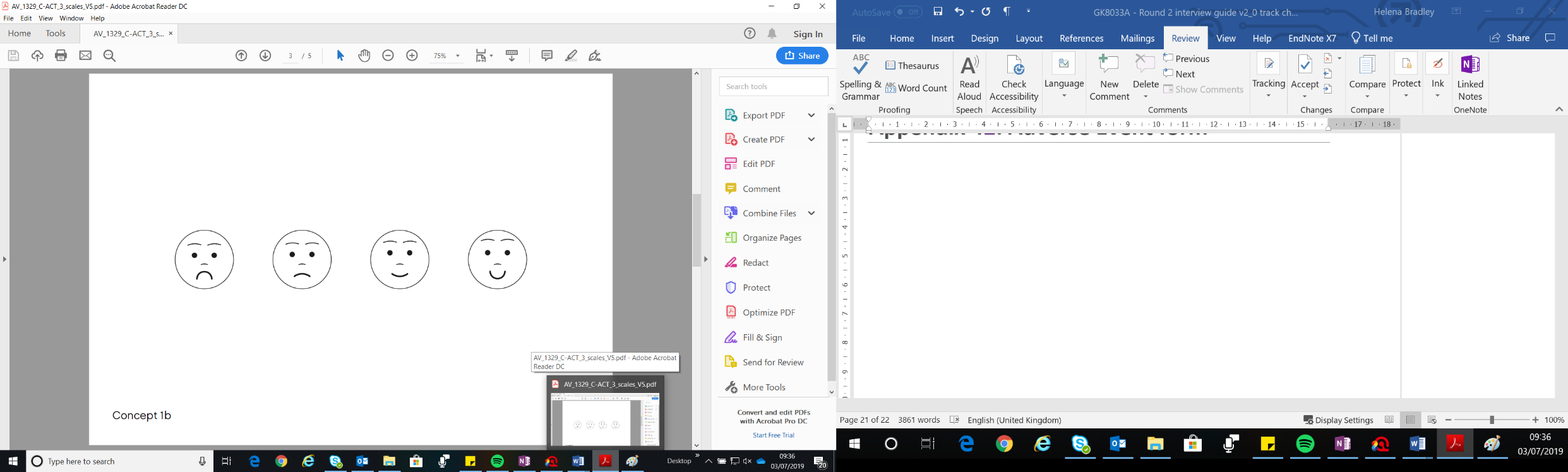

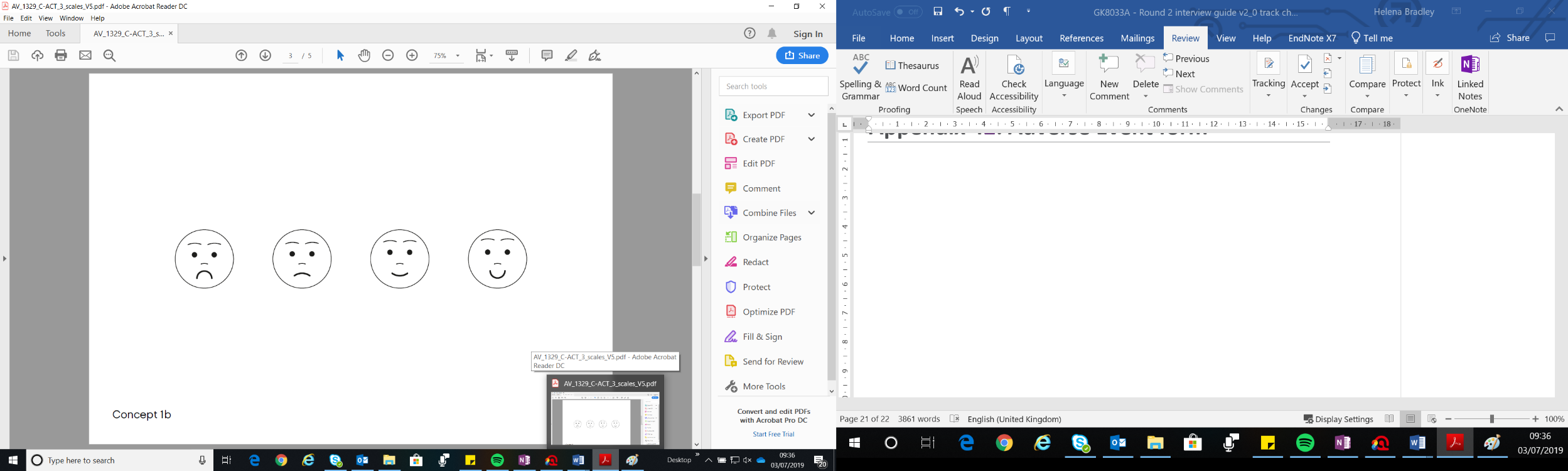

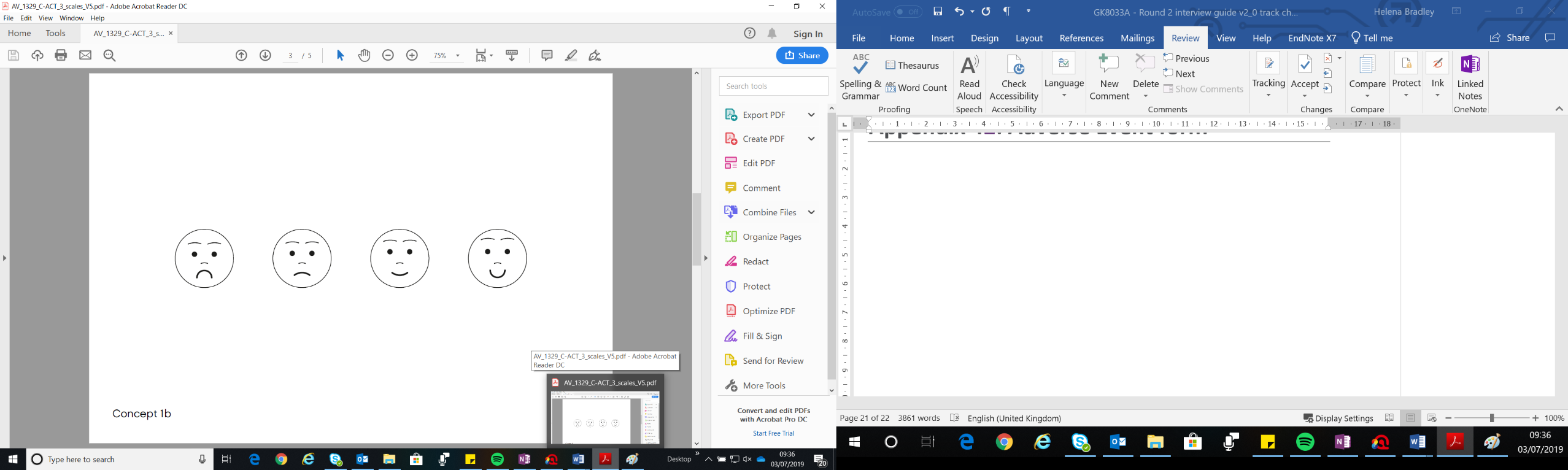

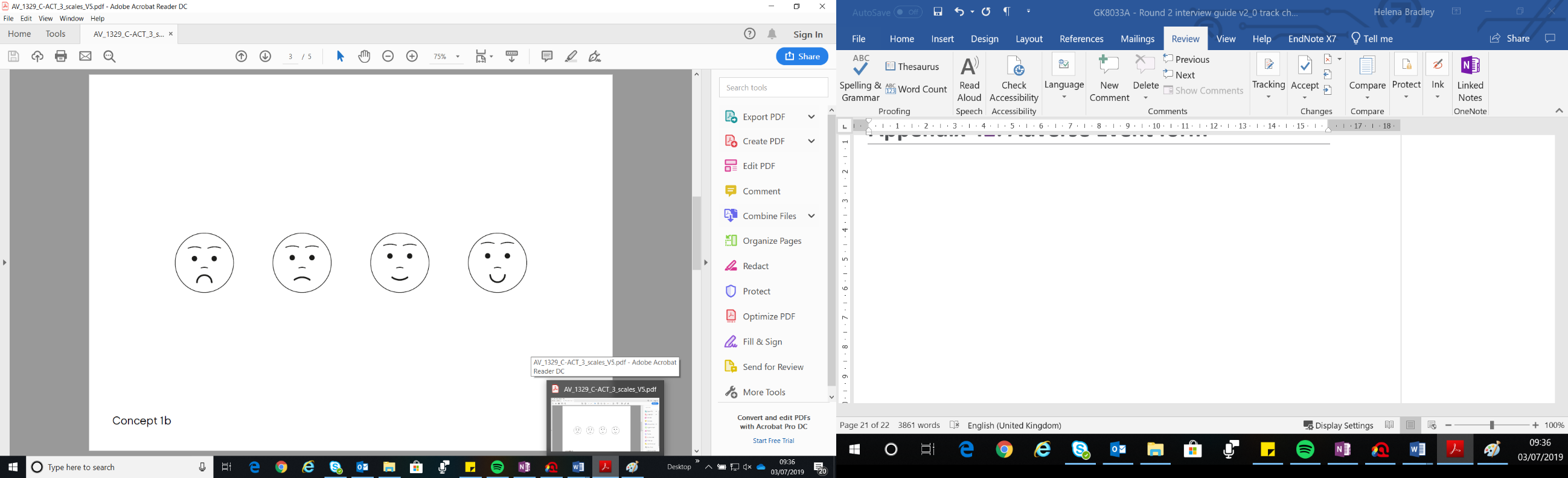


**Response scale B**

**
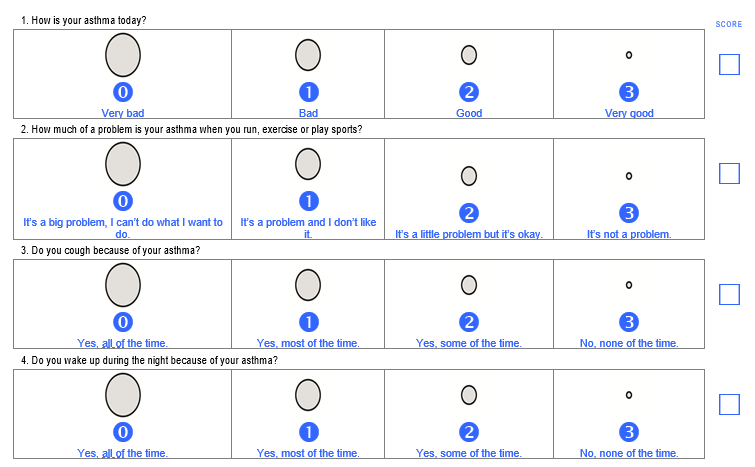
**

**Appendix 2: Copy of the current C-ACT**


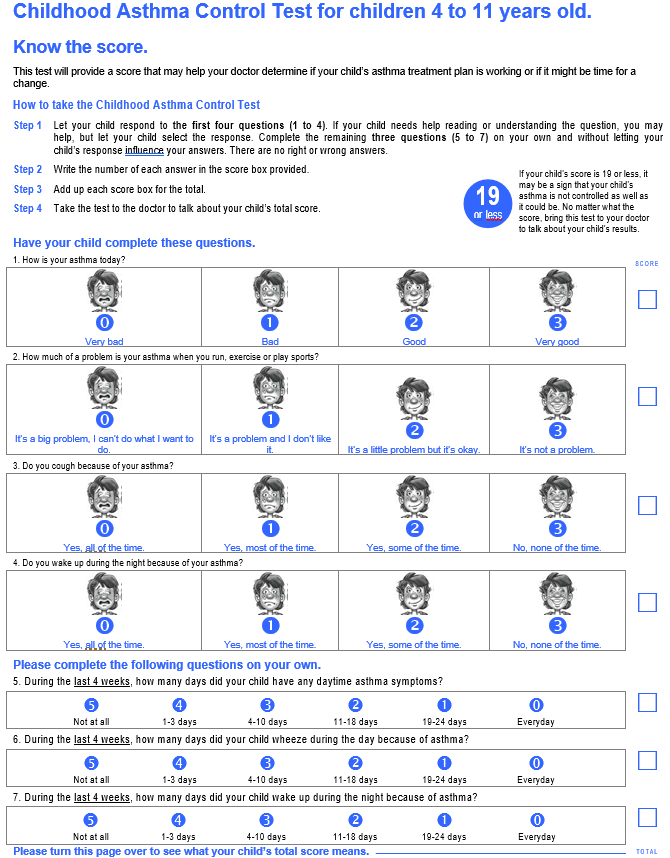


**Appendix 3: Additional questions for parent/caregiver**

The C-ACT is a questionnaire that is used to assess asthma control in children ages 4-11 years old and has been approved for use in medical practice. This research study is being done to test new response option images, to be included in the Childhood Asthma Control Test (C-ACT).

1. **Which new response option images do you think will be better for children to use to report their asthma severity?**

Simple faces  _1_


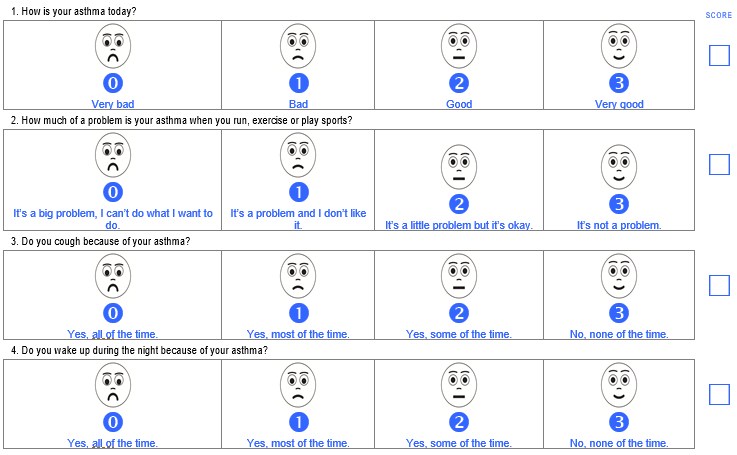

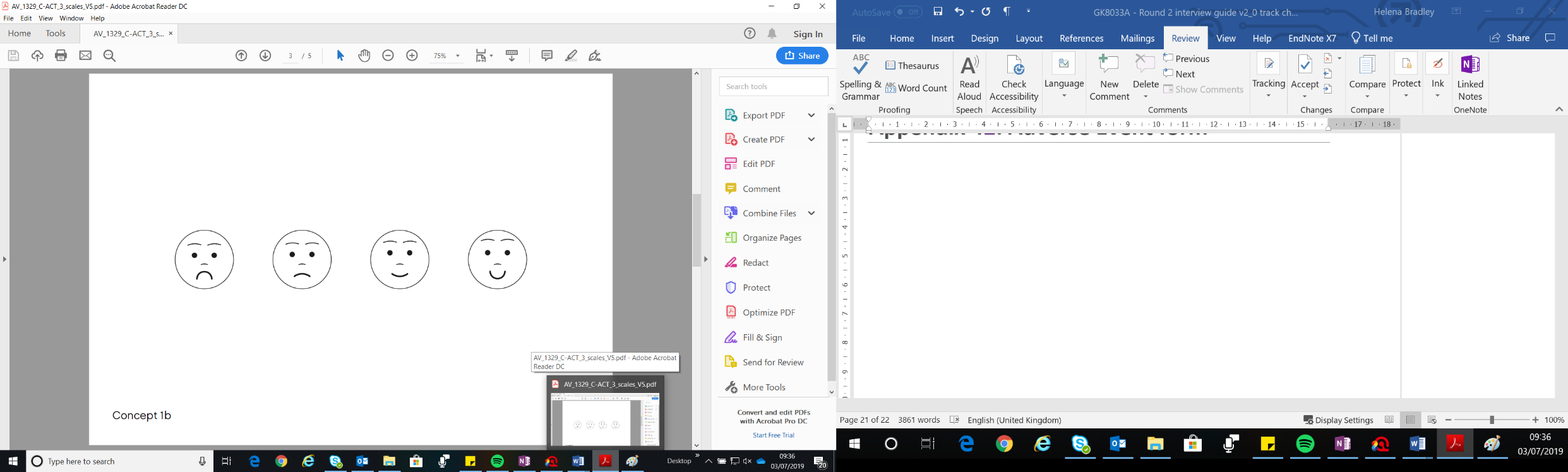

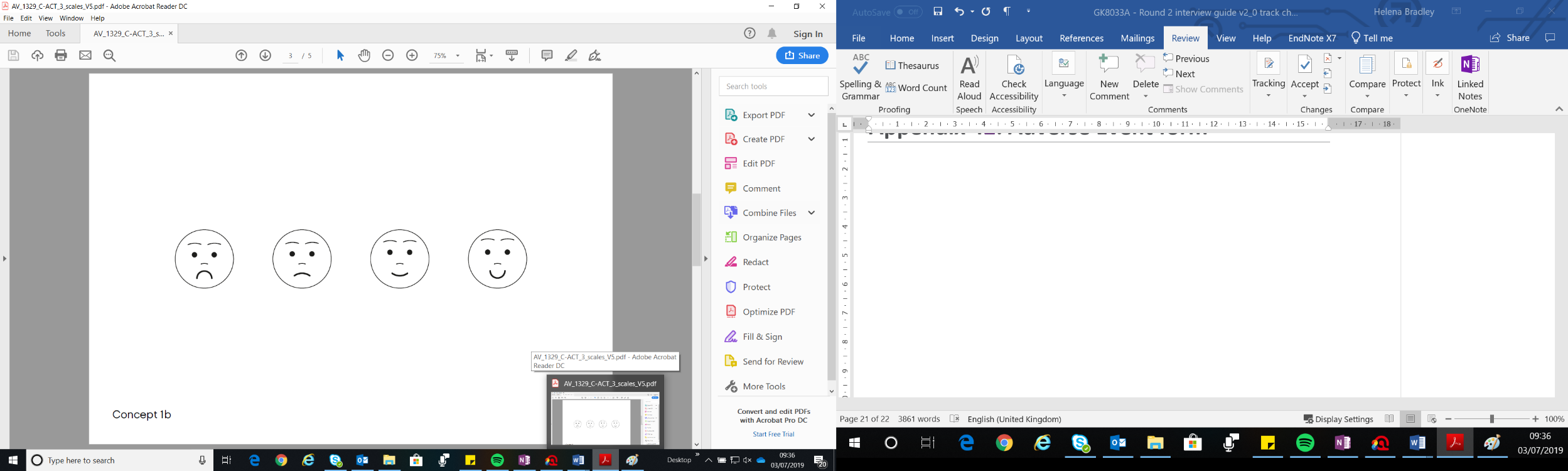

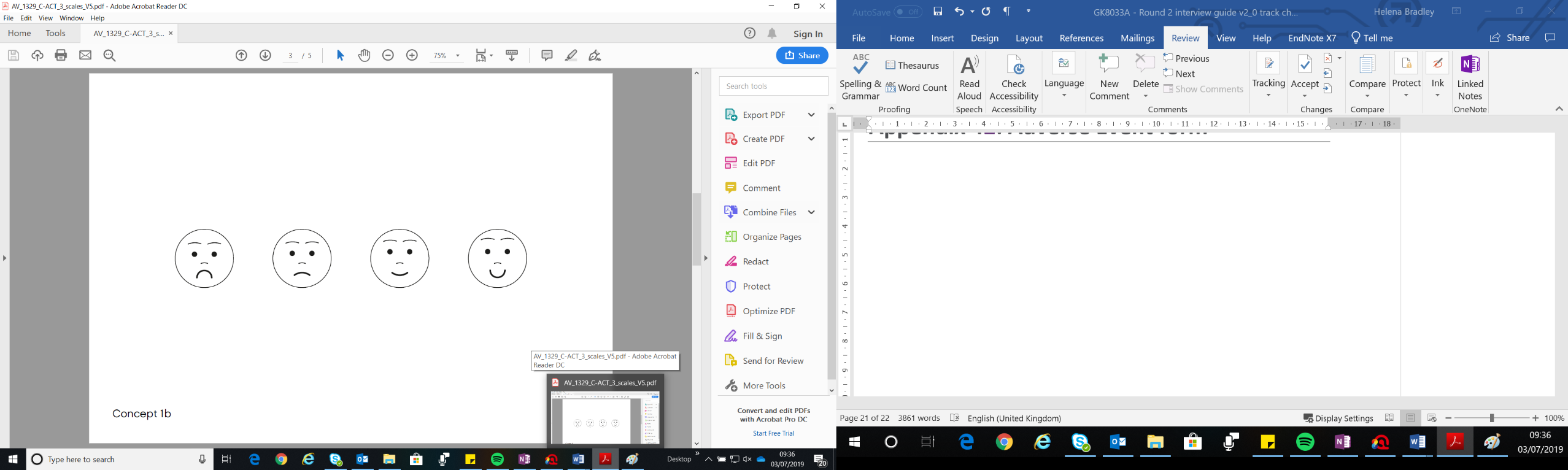

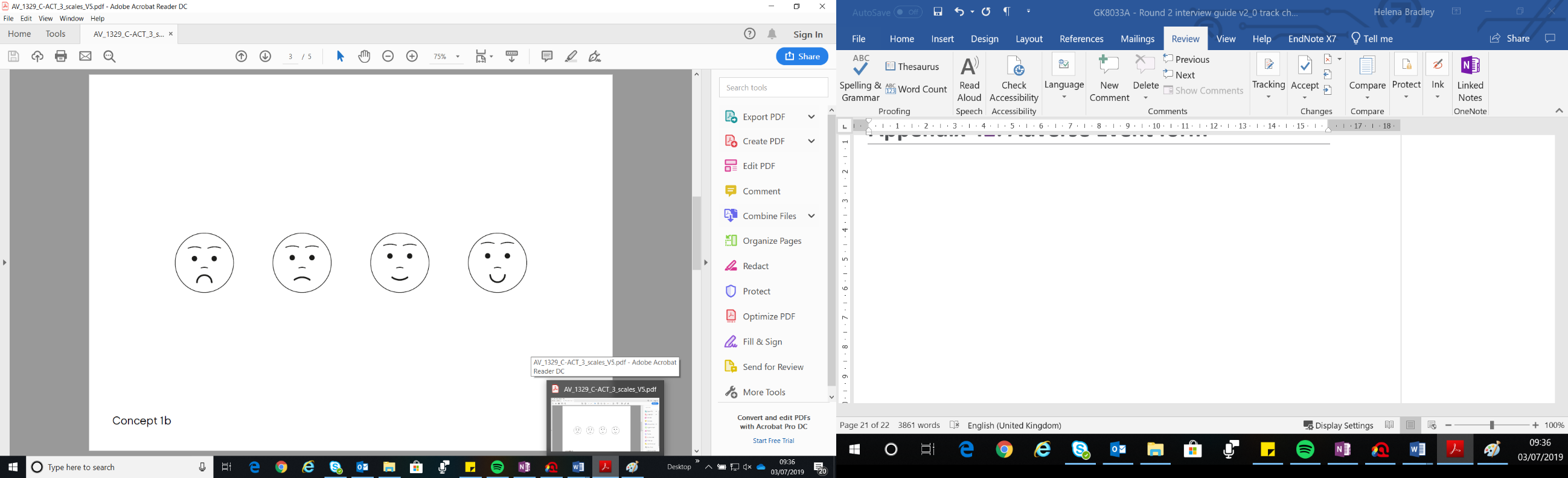


Circles of decreasing size _2_


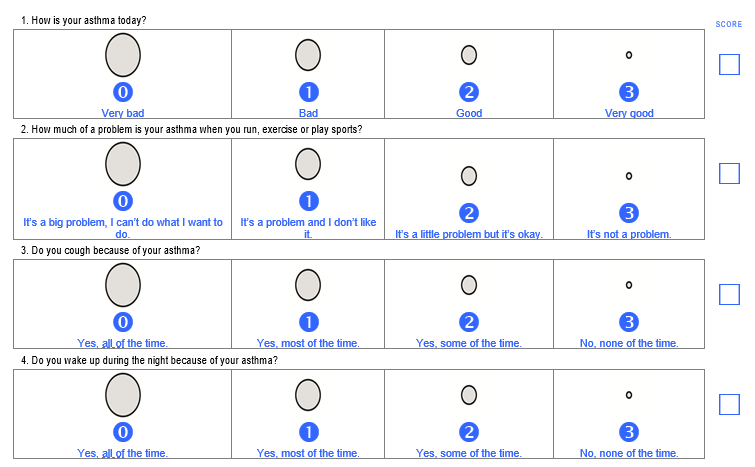


**Why?**

_______________________________________________________________________________________________________________________________________________________________________________________________________________________________________________________________________________________________________________________________________________________________________

1. **For the simple faces, do you think your child will be able to relate to these faces?**

Yes _1_

No _2_

**Why? Would you suggest changing the faces in any way to make them more relatable for your child?**

_______________________________________________________________________________________________________________________________________________________________________________________________________________________________________________________________________________________________________________________________________________________________________

**Appendix 4: Adverse Event form**


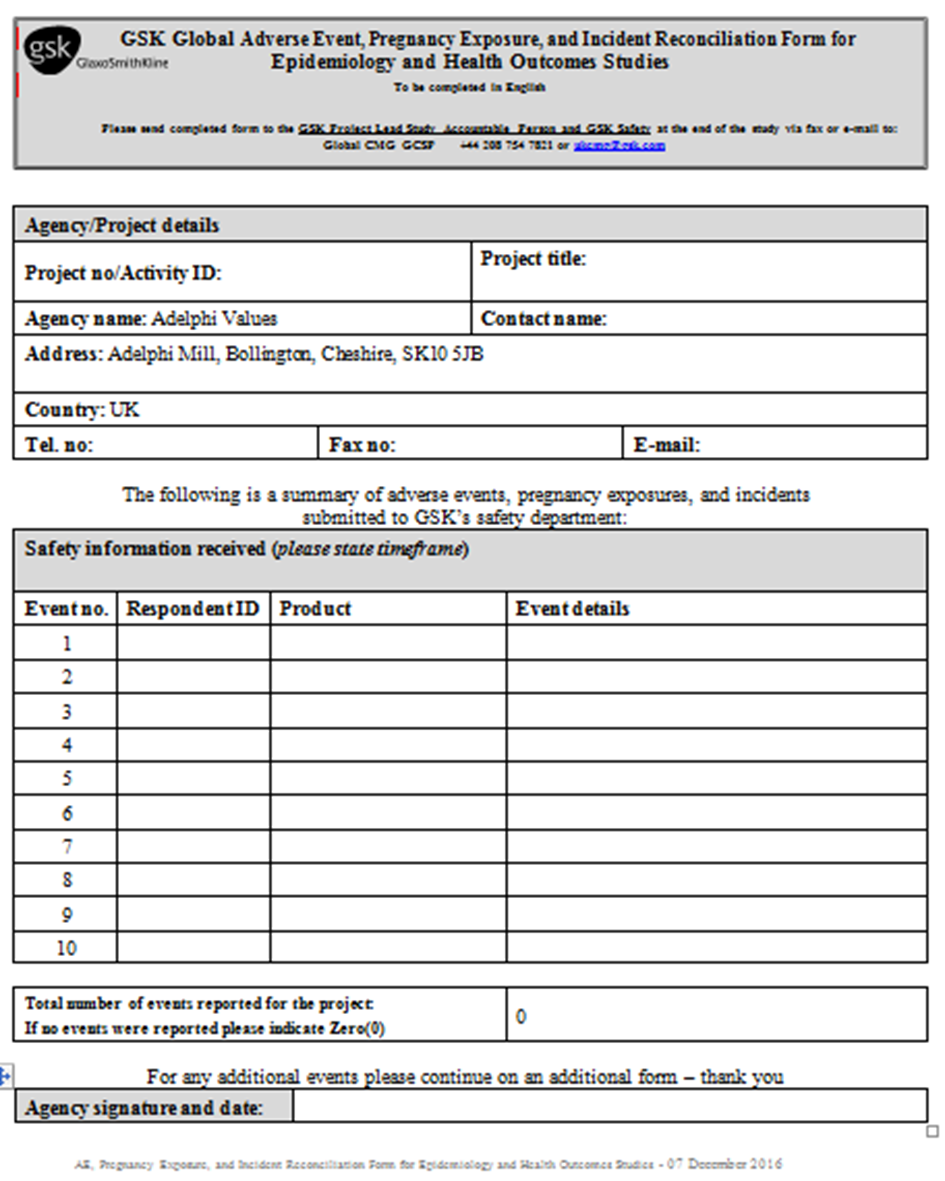


**Supplementary references**

1. Oros LB, de Minzi MCR. A review study of psychometric functioning of a picture scale to assess joy in childhood (2015) Psychology 6(03):223.

2. Baggott C, Baird J, Hinds P, Ruland CM, Miaskowski C. Evaluation of Sisom: A computer-based animated tool to elicit symptoms and psychosocial concerns from children with cancer (2015) Eur J Oncol Nurs 19(4):359-369.

3. Bieri D, Reeve RA, Champion GD, Addicoat L, Ziegler JB. The Faces Pain Scale for the self-assessment of the severity of pain experienced by children: development, initial validation, and preliminary investigation for ratio scale properties (1990) Pain 41(2):139-150.

4. de Castro Gonçalves J, Mónica Oliveira A, Cunha Batalha L, Fernandes AM, Viegas R, Duarte Silva A. A functional measurement approach to the Children’s Anxiety and Pain Scale-CAPS: contributions to its construct validity (2014) Psicológica 35(3): 653-674.

5. Chambers CT, Giesbrecht K, Craig KD, Bennett SM, Huntsman E. A comparison of faces scales for the measurement of pediatric pain: children's and parents' ratings (1999) Pain 83(1):25-35.

6. Muris P, Meesters C, Mayer B, Bogie N, Luijten M, Geebelen E, et al. The Koala Fear Questionnaire: a standardized self-report scale for assessing fears and fearfulness in pre-school and primary school children (2003) Behav Res Ther 41(5):597-617.

7. Victorine A, Wong Z, Geilani J, Charters A. Teddy Bear Hospital (TBH)–reducing children’s fear of doctors and hospital environment (2002) Pediatrics 110(3):e33.

8. Roth I. Mayo Clinic Minute: How emojis help cancer treatment, research. Available: https://newsnetwork.mayoclinic.org/discussion/mayo-clinic-minute-how-emojis-help-cancer-treatment-research/. Accessed January 2021.

9. Buchanan H. Development of a computerised dental anxiety scale for children: validation and reliability (2005) Br Dent J 199(6):359.

10. Farooqui N, Phillips G, Barrett C, Stukus D. Acceptability of an interactive asthma management mobile health application for children and adolescents (2015) Ann Allergy Asthma Immunol 114(6):527-529.

11. Christie M, French D, Weatherstone L, West A. The patients' perceptions of chronic disease and its management: Psychosomatics, holism and quality of life in contemporary management of childhood asthma (1991) Psychother Psychosom 56(4):197-203.

12. Christie MJ, French D, Sowden A, West A. Development of child-centered disease-specific questionnaires for living with asthma (1993) Psychosom Med 55(6):541-548.

13. Rebok G, Riley A, Forrest C, Starfield B, Green B, Robertson J, et al. Elementary school-aged children's reports of their health: a cognitive interviewing study (2001) Qual Life Res 10(1):59-70.

14. McGrath PJ, Pianosi PT, Unruh AM, Buckley CP. Dalhousie dyspnea scales: construct and content validity of pictorial scales for measuring dyspnea (2005) BMC Pediatrics 5(1):33.

15. Wong DL, Baker CM. Pain in children: comparison of assessment scales (1988) Pediatr Nurs 14(1):9-17.

16. Orcesi S, Ariaudo G, Mercuri E, Beghi E, Rezzani C, Balottin U, et al. A new self-report quality of life questionnaire for children with neuromuscular disorders: presentation of the instrument, rationale for its development, and some preliminary results (2014) J Child Neurol 29(2):167-181.

17. Hicks CL, von Baeyer CL, Spafford PA, van Korlaar I, Goodenough B. The Faces Pain Scale – Revised: toward a common metric in pediatric pain measurement (2001) Pain 93(2):173-183.

18. Borgers N, Sikkel D, Hox J. Response effects in surveys on children and adolescents: The effect of number of response options, negative wording, and neutral mid-point (2004) Qual Quant 38(1):17-33.

19. Global Initiative for Asthma. Global Strategy for Asthma Management and Prevention (2020). Available from: https://ginasthma.org/gina-reports/. Accessed January 2021.
